# The Prognostic Value of Multiple Systemic Inflammatory Biomarkers in Preoperative Patients With Non-small Cell Lung Cancer

**DOI:** 10.3389/fsurg.2022.830642

**Published:** 2022-04-04

**Authors:** Kai Wang, Qidi Zhao, Tao Yan, Deyu Guo, Jichang Liu, Guanghui Wang, Jiajun Du

**Affiliations:** ^1^Institute of Oncology, Shandong Provincial Hospital, Cheeloo College of Medicine, Shandong University, Jinan, China; ^2^Department of Healthcare Respiratory Medicine, Shandong Provincial Hospital, Cheeloo College of Medicine, Shandong University, Jinan, China; ^3^Department of Thoracic Surgery, Shandong Provincial Hospital, Cheeloo College of Medicine, Shandong University, Jinan, China

**Keywords:** non-small cell lung cancer (NSCLC), inflammatory biomarkers, nutrient biomarkers, prognostic model, nomogram, albumin-to-globulin ratio (AGR), aspartate transferase-to-neutrophil ratio index (ANRI), basophil-to-lymphocyte ratio (BLR)

## Abstract

**Introduction:**

The preoperative inflammatory and nutrient status of the patient are closely correlated to the outcome of surgery-based treatment for non-small cell lung cancer (NSCLC). We aimed to investigate the prognostic value of inflammation and nutrient biomarkers in preoperative patients with non-small cell lung cancer (NSCLC) by constructing a prognostic predictive model.

**Methods:**

We retrospectively studied 995 patients with NSCLC who underwent surgery in the Shandong Provincial Hospital and randomly allocated them into the training and validation group with a ratio of 7:3. We then compared their prognostic performance and conducted univariate Cox analyses with several clinicopathological variables. Based on the performance of the receiver operating characteristic (ROC) curves and decision curves analysis (DCA), the prognostic model was optimized and validated.

**Result:**

The median overall overall survival (OS) of patients was 74 months. Univariate Cox analysis indicated that fifteen inflammatory biomarkers were significantly correlated with OS (*p* < 0.100). Multivariate Cox analysis revealed that the model incorporating grade, age, stage, basophil-to-lymphocyte ratio (BLR, ≥0.00675 vs. < 0.00675) and albumin-to-globulin ratio (AGR, ≥1.40 vs. <1.40) showed the maximum area under the curve (AUC, 0.744). The C-index in the training and validation group was 0.690 and 0.683, respectively. The 3-year integrated discrimination improvement (IDI) compared to TNM (Tumor Node Metastasis) stage was 0.035 vs. 0.011 in the training and validation group, respectively.

**Conclusions:**

Lower AGR, ANRI, and higher BLR were associated with a worse outcome for patients with NSCLC. We constructed a prognostic nomogram with risk stratification based on inflammatory and nutrient biomarkers. The discrimination and calibration abilities of the model were evaluated to confirm its validity, indicating the potential utility of this prognostic model for clinical guidance.

## Introduction

Lung cancer is still the most lethal malignancy in the world, accounting for the highest cancer-related mortality of 18% for both genders in 2020 (1). Approximately 85% of the patients can be classified as non-small cell lung cancer (NSCLC), while the majority of histological subtypes are lung adenocarcinoma (LUAD) and lung squamous cell carcinoma (LUSC) (2). For patients with early-stage and localized advanced lung carcinoma, surgery is the primary therapy and the only effective means of treatment (3, 4). Although the early diagnosis and treatment modalities against NSCLC have been progressing rapidly in the past few decades (5, 6), the prognosis of patients remains unfavorable, with a 5-year survival of 10 to 20% worldwide (1, 7, 8). Meanwhile, the accurate prediction of clinical outcomes for patients with NSCLC remains a challenge for clinicians.

Systemic inflammation has a confirmed correlation with tumorigenesis (9). Inflammatory cells, together with chemokines and cytokines derived from the inflammatory response, are important constituents of the tumor microenvironment (TME) in the tumorous tissues (10). These inflammatory mediators and cellular effectors could facilitate tumor progression and metastasis in many ways, such as altering responses to chemotherapy drugs, promoting angiogenesis, and inhibiting adaptive immune responses (11, 12). Egeblad and colleagues found that in mouse models, sustained lung inflammation could promote metastasis of cancer cells (13). A sustained inflammatory stimulus can lead to the formation of neutrophil extracellular traps (NETs), which results in the activation of the integrin α3β1 signaling and consequently the enhanced proliferation of dormant cancer cells (13). Reciprocally, cancer cells can act on the inflammatory cells to escape immune clearance and surveillance.

Nutrients also closely correlate to tumorigenesis. Most of the tumor cells are accompanied by increased energy consumption and enhanced biosynthesis during the process of proliferation (14), which is a remarkable hallmark of cancer (15). Numerous nutrients can influence the metabolism of tumor cells by regulating the expression of oncogenes, affecting cell differentiation, and exerting inflammation-associated effects (16). In response to these nutrients, corresponding transcription factors and signaling pathways are activated, contributing to the tumor development and progression (17). Under amino acid-abundant conditions, the mTORC1 pathway could be activated and stimulate the proliferation-promoted metabolism of tumor cells (18). Besides the regulation of cellular processes at the post-transcriptional level, nutrients could also have a significant impact on the expression of key genes, such as by altering the methylation status of promoter regions, which lead to the alteration of DNA structure (19). Consequently, we believe that nutrient biomarkers, which could indicate the systemic nutrient status, could also be associated with the prognosis of NSCLC patients.

Preoperative inflammatory and nutrient status, which could be reflected in paraneoplastic symptoms (e.g., pyrexia, diaphoresis, and weight loss) and systemic inflammatory and nutrient-associated biomarkers, could significantly predict the prognosis of patients with NSCLC. These biomarkers have been widely studied in preoperative patients with malignancies (20, 21). In this study, we aimed to assess the prognostic effects of multiple systemic inflammatory and nutrient biomarkers for OS and PFS (progression-free survival), enumerate and evaluate their different combinations with other clinicopathological variables for individual prognostic prediction.

## Methods

### Patients and Clinicopathological Characteristics

We consecutively collected information for patients with NSCLC from the Thoracic Surgery Department, Shandong Provincial Hospital between January 2006 and December 2016. The demographic and clinicopathological information of patients contained age at diagnosis, gender, laterality, smoking history, primary site, grade, histology, scope of surgery, tumor size, infiltrating extents, lymph node and tumor metastasis, adjuvant therapy (including chemo/radiotherapy/targeted therapy), surgical methods (by video-assisted thoracoscopic surgery (VATS) or thoracotomy). The smoking index was computed as the daily mean consumption of cigarettes × smoking time (year). The axillary temperature in perioperative period were measured at least twice a day and recorded on the temperature charts. The stage of patients was transformed into the eighth edition TNM (Tumor Node Metastasis) stage classification. We also collected the blood cell/serum indices of preoperative patients from their blood biochemical and routine tests, containing levels of aspartate aminotransferase (AST, U/L), alanine aminotransferase (ALT, U/L), albumin (Alb, g/L), globulin (Glo, g/L), fibrinogen (Fib, g/L), gamma-glutamyl transferase (GGT, U/L), monocyte (M, 10^9^/L), lymphocyte (L, 10^9^/L), neutrophil (N, 10^9^/L), eosinophil (E, 10^9^/L), basophil (B, 10^9^/L) and platelet (PLT, 10^9^/L).

The patients were regularly followed up by telephone after being discharged. The follow-up schedule was designed as previously described (22). The inclusion criteria included (1) specific thoracic surgery were performed, (2) histology were adenocarcinoma and squamous cell carcinoma (confirmed by pathological report), (3) primary and (4) unilateral carcinoma. The exclusion criteria included (1) overall survival ≤ 1 (month), (2) incomplete variables mentioned above, (3) underwent neoadjuvant therapy, (4) combined with other malignant carcinoma. Then patients were randomly allocated into training and validation group at a ratio of 7:3. The primary outcome was overall survival (OS), indicating the time period (months) from the surgery to death for any reason or last time of follow-up. The secondary outcome was progression-free survival (PFS), indicating the time period (months) between surgery and tumor progression or death for any reason.

We obtained ethical approval from Biomedical Research Ethic Committee of Shandong Provincial Hospital. (SWYX:NO. 2021-435) The study complied with the World Medical Association Declaration of Helsinki.

### Process of Biomarkers

We calculated continuous biomarkers as following equations: the lymphocyte-associated inflammatory biomarkers including NLR (neutrophil-to-lymphocyte ratio) = N/L, PLR (platelet-to-lymphocyte ratio) = PLT/L, BLR (basophil-to-lymphocyte ratio) = B/L, MLR (monocyte-to-lymphocyte ratio)=M/L, SIRI (systemic inflammation response index) = N × M/L, SII (systemic immune inflammation index) = N × PLT/L; the albumin-associated nutrient biomarkers including AGR (albumin-to-globulin ratio) = A/G, AFR (albumin-to-fibrinogen ratio) = Alb/Fib, PNI (prognostic nutritional index) = serum albumin (g/L) + 5 × total lymphocytes count (/L). We also introduced some nutrient biomarkers which are associated with other systemic disease (such as liver cirrhosis and hepatocellular carcinoma), to access the potential interaction of NSCLC on systemic organs. These GGT- or AST-associated biomarkers included GAPI (glutamyl transpeptidase (GGT)-to-platelet ratio) = GGT/PLT, GLR (GGT-to-lymphocyte ratio) = GGT/L, ALRI (aspartate transferase (AST)-to-lymphocyte ratio) = AST/L, ANRI (aspartate transferase (AST-to-neutrophil ratio) = AST/N, APRI (AST-to-platelet ratio) = AST/PLT, FIB-4 score (fibrosis index based on four factors) = age (year) × AST/(PLT × ALT^1/2^). By ROC curves and the optimal cutoff values according to their maximum Youden index (sensitivity + specificity-1), the continuous variables were classified into categorical variables in the training group.

For SIS (systemic inflammation score) and NPS (neutrophil-platelet score), we adopted the most widely accepted cutoff values and classified methods in various cancers. SIS was defined as follows: patients with both <40 g/L serum albumin and <4.44 LMR were allocated to score 2; patients with both ≥40 g/L serum albumin and ≥4.44 LMR were allocated to score 0; the remaining patients were allocated to score 1 (20). NPS was defined as follows: patients with both >7.5 × 10^9^/L neutrophil and >400 × 10^9^/L platelet were allocated to score 2; patients with both ≤ 7.5 × 10^9^/L neutrophil and ≤ 400 × 10^9^/L platelet were allocated to score 0; the remaining patients were allocated to score 1 (21). We identified the classified method of F-NLR (fibrinogen-NLR score) by calculating the cutoff of fibrinogen and NLR (see below), respectively. F-NLR was defined as follows: patients with both Fib and NLR ≥ cutoff value were allocated to score 2; patients with both Fib and NLR < cutoff value were allocated to score 0; the remaining patients were allocated to score 1.

Specially, the cutoff value of smoking index was calculated by patients without non-smokers (smoking index = 0) considering its clinical significance. The smoking index was transformed into tripartite variables as non-smokers (0), low-level smokers (< cutoff value) and high-level smokers (>cutoff value). The variable pyrexia was derived from the maximun body temperature. We defined the pyrexia as pyrexia before surgery (≥37.3°C), hyperpyrexia before surgery (≥38°C), pyrexia after surgery (≥37.3°C) and hyperpyrexia after surgery (≥38°C).

### Statistical Analysis

Univariate Cox analyses of OS for clinicopathological variables and inflammatory and nutrient biomarkers were employed in the training group to preliminarily identify the prognostic factors. To avoid including the repeating blood cell/serum indices, we listed all potential combinations of inflammatory biomarkers which achieved significance in the univariate Cox analyses. Each combination were included in the multivariate Cox analysis together with significant clinicopathological variables in a forward stepwise manner. Variables of significance in the multivariate Cox analyses were eligible for the construction of the prognostic model. Based on the result, different prognostic models were constructed and validated by comparing the receiver operating characteristic (ROC) curves, with their time-dependent area under curve (AUC), decision curve analysis (DCA), integrated discrimination improvement (IDI) and net reclassification index (NRI) to the TNM staging system.

Following the validation, we finalized the prognostic model by comprehensive evaluation, including exhibiting the maximum AUC, concordance index (C-index) and time-dependent (3-year) IDI. We broke down the ratio of inflammatory biomarkers in the final prognostic model into separate blood cell or serum indices, and included them into univariate and multivariate analysis with same clinicopathological variables (as previously indicated), and then calculated the time-dependent AUC and C-index to compare the pros and cons of these models (model with inflammatory biomarkers, model with blood cell or serum indices, model with clinicopathological variables only and model of TNM stage). Risk stratification was generated to divide patients into low-risk, intermediate-risk and high-risk groups.

The C-index was calculated to assess the accuracy of the model's prediction. To evaluate the collinear performance of the model, all-subsets regression analyses were carried out both in the training and validation group. In addition, we measured the different model indices including Akaike information criterion (AIC), Bayesian information criterion (BIC), Nagelkerke *R*-Square and root mean squared error (RMSE), to compare the prognostic performance between TNM staging system and prognostic model.

In order to exclude the influence of confounding factors on the choice of model variables, we performed subgroup analysis for surgical scope (sublobectomy, lobectomy, extended lobectomy and pneumonectomy) and TNM staging system (I, II, III and IV). We grouped all patients according to different surgical scopes or TNM stages, and analyzed them according to the same method. Time-dependent (3-year) AUC and C-index of each model were calculated to evaluate their prognostic performance, and to identify the inflammatory factors most associated with the prognosis of NSCLC patients.

The hazard ratio (HR) was calculated with *p*-value and 95% confidence interval (CI) in the training group. Variables with a *p*-value < 0.100 were considered significant in the univariate Cox analysis, and less than 0.050 in the multivariate Cox analysis. Concordance index (C-index) was calculated by performing a resampling (1,000 bootstrap) based on the two groups. Kaplan-Meier analyses and log-rank tests of different variables were performed in OS or PFS for comparing the survival difference.

Risk stratification was calculated by X-tile 3.6.1 (Yale University, New Haven, CT, USA) based on the total points of each patients in the training group. All statistical analyses were completed by SPSS (26.0), R environment (4.1.0) and Rstudio (1.4.1717).

## Results

### Characteristics of Patient

**Figure 1:**
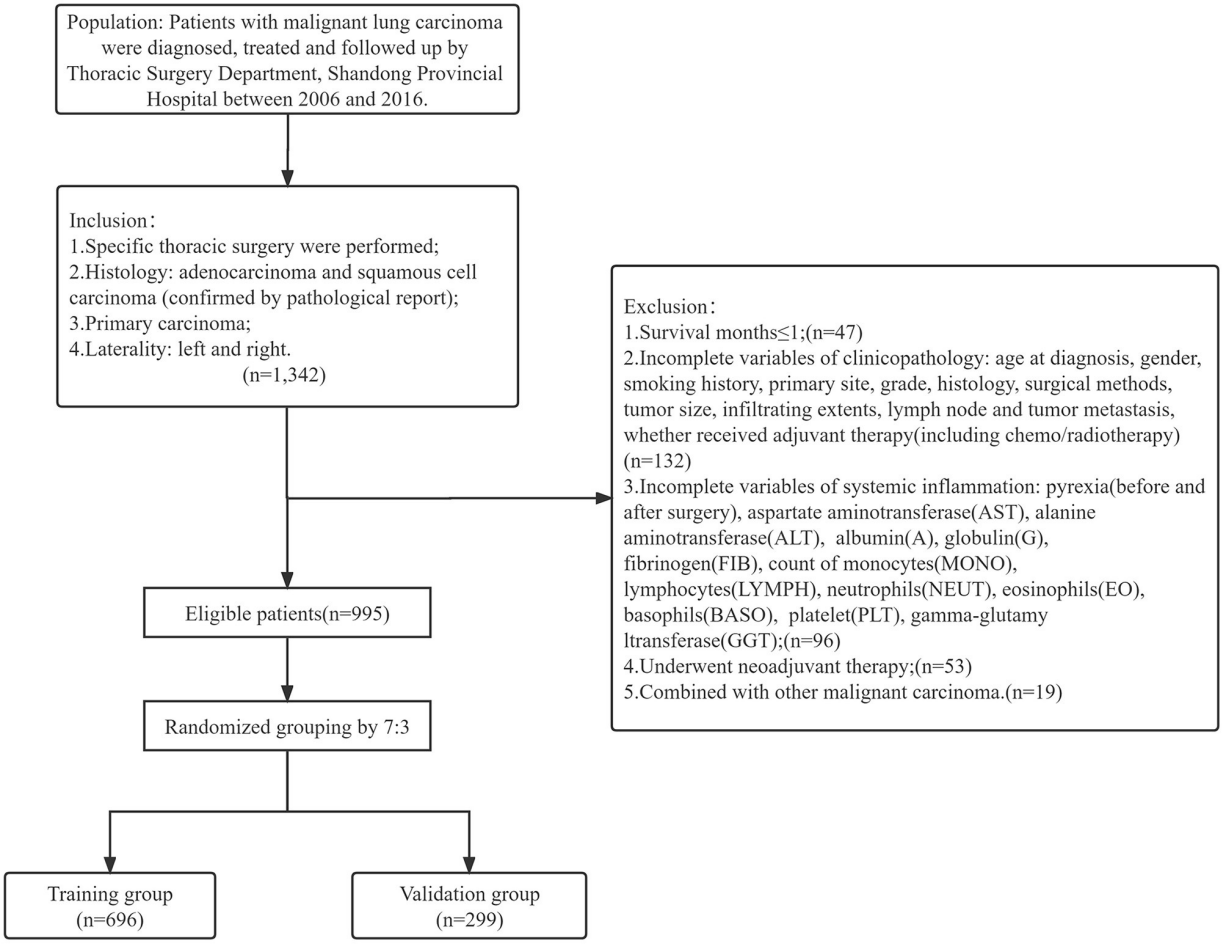
Inclusion and exclusion criteria for training and validation group.

A total of 1,342 patients with clinical information were collected. Among them, 995 patients who conformed to the inclusion and exclusion criteria were ultimately included in the study. We grouped these patients into a randomized training group (*n* = 696) and validation group (*n* = 299) according to a 7:3 ratio (Figure 1).

The baseline information are shown in Table 1. The population of patients were composed of Asian (Chinese), aged from 27 to 84 years. The scope of surgery was mainly lobectomy (665, 66.8%), while others were sublobectomy (120, 12.1%), extended lobectomy (112, 11.3%) and pneumonectomy (98, 9.8%), respectively. For the detailed classification of the surgical scope, please refer to the annotation in Table 1. There were 814 patients (81.8%) underwent traditional thoracotomy, and 181 patients underwent video-assisted thoracoscopic surgery (VATS). Of the total patients, 667 (67.0%) of them received single or multiple forms of adjuvant therapy, including but not limited to chemotherapy, radiotherapy and targeted therapy. The median survival time of OS was 74 months, and 54 months of PFS in total patients (Supplementary Figure 1). There were 510 (51.3%) events of OS and 514 (51.7%) events of PFS occurred during the whole follow up time. The 1-year, 3-year and 5-year OS were 88.9, 61.2, and 52.3%; and 1-year, 3-year and 5-year PFS were 75.3%, 54.6% and 48.8%, respectively. Chi-square tests (or Fisher's exact tests) between training and validation group illustrated the well-independence of two groups (Table 1).

**Table 1:** The baseline information of population in total patients, training group, and validation group.

**Characteristics**	**Total patients (*n* = 995)**	**Training group (*n* = 696)**	**Validation group (*n* = 299)**	***P*-value^**a**^**
	***n* (%)**	***n* (%)**	***n* (%)**	
**Histology**				
Adenocarcinoma	528 (53.1)	364 (52.3)	164 (54.8)	0.460
Squamous cell carcinoma	467 (46.9)	332 (47.7)	135 (45.2)	
**Gender**				
Male	707 (71.1)	500 (71.8)	207 (69.2)	0.406
Female	288 (28.9)	196 (28.2)	92 (30.8)	
**Age**				
≥63	418 (42)	291 (41.8)	127 (42.5)	0.846
<63	577 (58)	405 (58.2)	172 (57.5)	
**Primary site**				
Upper lobe	489 (49.1)	348 (50)	141 (47.2)	0.739
Middle lobe	54 (5.4)	34 (4.9)	20 (6.7)	
Lower lobe	357 (35.9)	246 (35.3)	111 (37.1)	
Hilus of the lung	47 (4.7)	33 (4.7)	14 (4.7)	
Overlapping lesion of lung	48 (4.8)	35 (5)	13 (4.3)	
**Laterality**				
Left	475 (47.7)	329 (47.3)	146 (48.8)	0.652
Right	520 (52.3)	367 (52.7)	153 (51.2)	
**Grade**				
Well-differentiated	89 (8.9)	63 (9.1)	26 (8.7)	0.909
Moderately differentiated	629 (63.2)	442 (63.5)	187 (62.5)	
Poorly and undifferentiated	277 (27.8)	191 (27.4)	86 (28.8)	
**TNM stage**				
I	357 (35.9)	239 (34.3)	118 (39.5)	0.271
II	281 (28.2)	204 (29.3)	77 (25.8)	
III + IV	357 (35.9)	253 (36.4)	104 (34.8)	
**Scope of surgery** ^**b**^				
Sublobectomy	120 (12.1)	80 (11.5)	40 (13.4)	0.569
Lobectomy	665 (66.8)	463 (66.5)	202 (67.6)	
Extended lobectomy	112 (11.3)	84 (12.1)	28 (9.4)	
Pneumonectomy	98 (9.8)	69 (9.9)	29 (9.7)	
**Smoking index**				
0	359 (36.1)	244 (35.1)	115 (38.5)	
<387.5	86 (8.6)	61 (8.8)	25 (8.4)	0.591
>387.5	550 (55.3)	391 (56.2)	159 (53.2)	
**Adjuvant therapy** ^**c**^				
None	328 (33)	223 (32)	194 (64.9)	0.344
Yes	667 (67)	473 (68)	105 (35.1)	
**Pyrexia before surgery (≥37.3** **°** **C)**				
No	846 (85)	594 (85.3)	252 (84.3)	0.666
Yes	149 (15)	102 (14.7)	47 (15.7)	
**Pyrexia after surgery (≥37.3** **°** **C)**				
No	393 (39.5)	278 (39.9)	115 (38.5)	0.661
Yes	602 (60.5)	418 (60.1)	184 (61.5)	
**Hyperpyrexia before surgery (≥38** **°** **C)**				
No	949 (95.4)	665 (95.5)	284 (95)	0.698
Yes	46 (4.6)	31 (4.5)	15 (5)	
**Hyperpyrexia after surgery (≥38** **°** **C)**				
No	761 (76.5)	534 (76.7)	227 (75.9)	0.784
Yes	234 (23.5)	162 (23.3)	72 (24.1)	
**VATS** ^**d**^				
No	814 (81.8)	568 (81.6)	246 (82.3)	0.803
Yes	181 (18.2)	128 (18.4)	53 (17.7)	
**ANRI**				
≥4.91	531 (53.4)	373 (53.6)	158 (52.8)	0.828
<4.91	464 (46.6)	323 (46.4)	141 (47.2)	
**BLR**				
≥0.00675	642 (64.5)	457 (65.7)	185 (61.9)	0.252
<0.00675	353 (35.5)	239 (34.3)	114 (38.1)	
**AGR**				
≥1.40	591 (59.4)	421 (60.5)	170 (56.9)	0.285
<1.40	404 (40.6)	275 (39.5)	129 (43.1)	
**SII**				
≥572.205	427 (42.9)	304 (43.7)	123 (41.1)	0.458
<572.205	568 (57.1)	392 (56.3)	176 (58.9)	
**SIRI**				
≥1.155	437 (43.9)	311 (44.7)	126 (42.1)	0.459
<1.155	558 (56.1)	385 (55.3)	173 (57.9)	

*Annotation and abbreviation: a, Chi-square test; b, Sublobectomy, including partial/wedge/segmental/sleeve resection, lingulectomy and partial lobectomy; Extended lobectomy, including bilobectomy and lobectomy with pulmonary angioplasty; Pneumonectomy, including complete/total/standard pneumonectomy and radical/extended pneumonectomy; c, including chemotherapy and/or radiotherapy and/or targeted therapy. d, Video-assisted Thoracoscopic Surgery. ANRI, aspartate transaminase-to-neutrophil ratio index; BLR, basophil to lymphocyte ratio; AGR, albumin to-globulin ratio; SII, systemic immune-inflammation index; SIRI, systemic inflammation response index*.

### Confirmation of Variables

**Figure 2:**
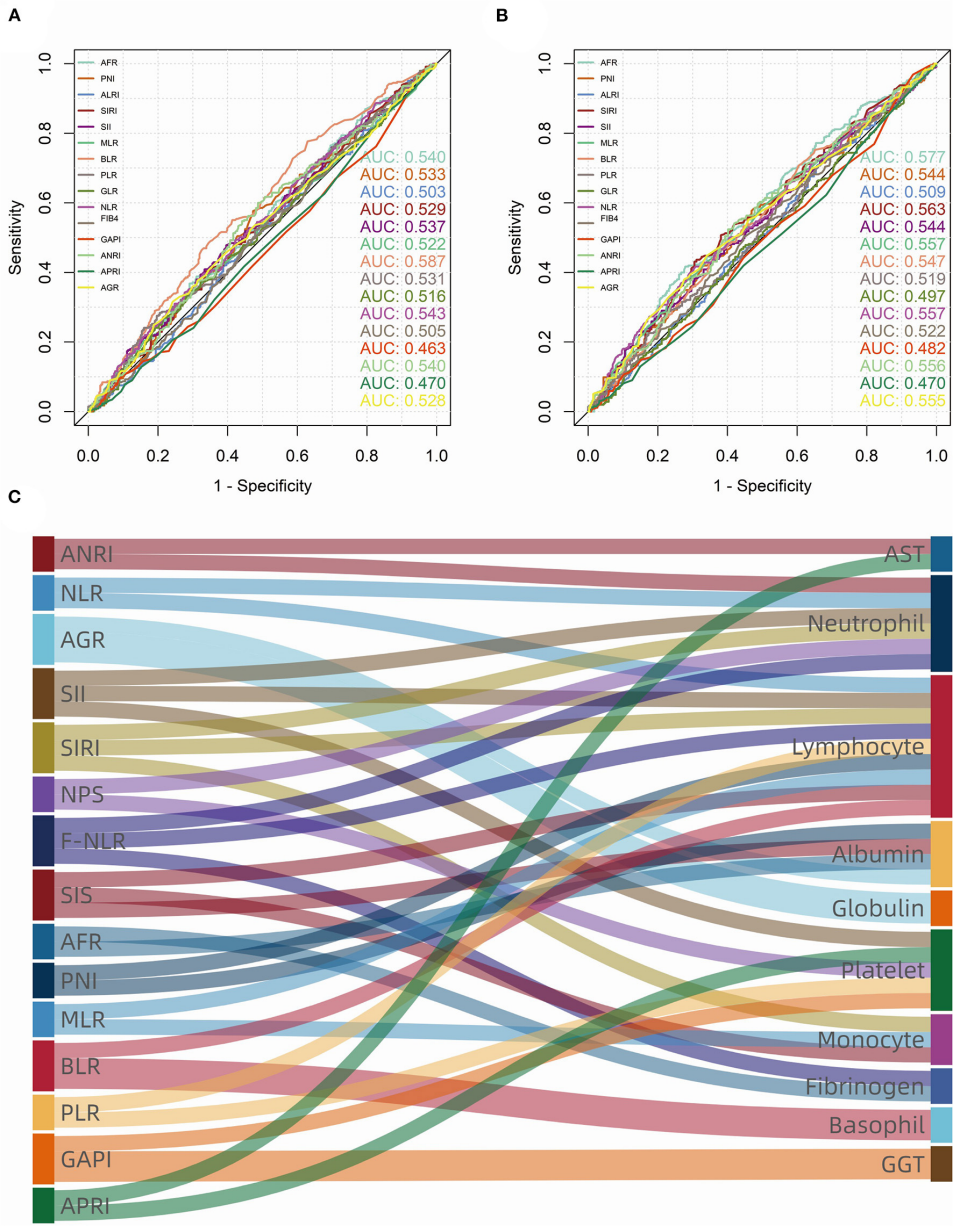
Receiver operating characteristic (ROC) curves and area under curve (AUC) of consecutive biomarkers in training group. **(A)** overall survival (OS); **(B)** progression-free survival (PFS). **(C)** The sankey diagram showed fifteen biomarkers (left) that were significant (*p* < 0.100) in univariate Cox analysis, which consist of ten blood cell/serum indices (right).

According to the maximum Youden index from ROC curves in the training group, the cutoff value of the continuous variables were calculated as follows: 2.745 (NLR), 38.365 (GLR), 168.745 (PLR), 0.00675 (BLR), 0.345 (MLR), 10.625 (AFR), 1.155 (SIRI), 0.123 (GAPI), 14.75 (ALRI), 4.91 (ANRI), 0.0721 (APRI), 572.21 (SII), 50.925 (PNI), 1.285 (FIB-4 score), 3.585 (Fib), 1.40 (AGR), 62.5 (age), and 387.5 (smoking index) (Figures 2A,B).

Univariate Cox analysis indicated that demographic and clinicopathological variables including gender, age, primary site, grade, stage, scope of surgery, smoking index, VATS (surgical methods) and adjuvant therapy were significant (*p* < 0.100) for OS (Table 2). The survival difference of fever patients was also concerned (Supplementary Figure 2). Interestingly, Kaplan-Meier analyses and log-rank tests showed that patients in the postoperative pyrexia group had a significantly (*p* < 0.050) better prognosis of PFS (D, *p* = 0.003). By contrast, pyrexia was not a significant prognostic factor for patients in other groups. What's more, univariate analysis of PFS showed differently that gender (*p* = 0.366) was not a significant prognostic factor, yet pyrexia after surgery (*p* = 0.004) still significantly indicated the low probability of tumor progression (Supplementary Table 1).

**Table 2:** Univariate and multivariate COX regression analysis of overall survival in training group.

**Characteristics**	**Univariate**		**Multivariate**	
	**Hazard ratio (95%CI)**	***P*-value**	**Hazard ratio (95%CI)**	***P*-value**
Histology (LUSC vs. LUAD)	1.118 (0.908–1.377)	0.293	-	-
Gender (female vs. male)	0.751 (0.59–0.955)	0.020	1.023 (0.709–1.476)	0.902
Age (<63 vs. ≥63)	0.635 (0.515–0.782)	<0.001	0.594 (0.476–0.742)	<0.001
**Primary site**				
Upper lobe	1	0.007	1	0.122
Middle lobe	0.772 (0.448–1.331)	0.353	1.121 (0.641–1.959)	0.690
Lower lobe	1.161 (0.927–1.455)	0.194	1.128 (0.896–1.42)	0.305
Hilus of the lung	1.831 (1.175–2.854)	0.008	1.34 (0.824–2.181)	0.238
Overlapping lesion of lung	0.554 (0.301–1.018)	0.057	0.518 (0.268–1)	0.050
Laterality (right vs. left)	1.075 (0.872–1.324)	0.500	-	-
**Grade**				
Well-differentiated	1	<0.001	1	0.037
Moderately differentiated	3.073 (1.757–5.373)	<0.001	2.077 (1.162–3.714)	0.014
Poorly and undifferentiated	3.707 (2.087–6.585)	<0.001	2.196 (1.202–4.012)	0.011
**TNM stage**				
I	1	<0.001	1	<0.001
II	2.125 (1.57–2.876)	<0.001	1.999 (1.436–2.781)	<0.001
III+IV	3.762 (2.85–4.965)	<0.001	3.404 (2.478–4.677)	<0.001
**Scope of surgery**				
Sublobectomy	1	0.094	1	0.564
Lobectomy	0.982 (0.704–1.37)	0.916	0.838 (0.595–1.181)	0.313
Extended lobectomy	1.192 (0.782–1.816)	0.413	0.93 (0.589–1.469)	0.757
Pneumonectomy	1.45 (0.947–2.219)	0.087	1.032 (0.642–1.658)	0.896
**Smoking index**				
0	1	0.006	1	0.724
<387.5	1.075 (0.706–1.639)	0.735	0.955 (0.587-1.552)	0.852
>387.5	1.44 (1.144–1.812)	0.002	1.102 (0.774-1.569)	0.590
Adjuvant therapy (yes vs. none)	1.287 (1.038–1.596)	0.022	0.992 (0.793-1.241)	0.945
Pyrexia before surgery (yes vs. no)	1.097 (0.819–1.468)	0.536	-	-
Pyrexia after surgery (yes vs. no)	0.918 (0.744–1.134)	0.429	-	-
Hyperpyrexia before surgery (yes vs. no)	1.362 (0.848–2.189)	0.201	-	-
Hyperpyrexia after surgery (yes vs. no)	1.153 (0.907–1.466)	0.245	-	-
VATS (yes vs. no)	0.543 (0.397–0.743)	<0.001	0.995 (0.7-1.414)	0.978
ANRI (<4.91 vs. ≥4.91)	1.473 (1.196–1.815)	<0.001	-	-
NLR (<2.745 vs. ≥2.745)	0.709 (0.574–0.876)	0.001	-	-
AGR (<1.40 vs. ≥1.40)	1.425 (1.156–1.757)	0.001	1.289 (1.031-1.612)	0.026
SII (<572.21 vs. ≥572.21)	0.768 (0.624–0.946)	0.013	-	-
SIRI (<1.155 vs. ≥1.155)	0.688 (0.558–0.847)	<0.001	-	-
**NPS**				
0	1	0.228	1	0.260
1	1.004 (0.694–1.452)	0.983	0.744 (0.501–1.103)	0.140
2	2.172 (0.898–5.257)	0.085	1.316 (0.523–3.315)	0.560
**F-NLR**				
0	1	<0.001	-	-
1	1.4 (1.089–1.8)	0.009	-	-
2	1.772 (1.376–2.283)	<0.001	-	-
**SIS**				
0	1	0.009	-	-
1	1.277 (0.989–1.648)	0.061	-	-
2	1.562 (1.173–2.079)	0.002	-	-
AFR (<10.625 vs. ≥10.625)	1.636 (1.324–2.02)	<0.001	-	-
PNI (<50.925 vs. ≥50.925)	1.382 (1.119–1.706)	0.003	-	-
ALRI (<14.75 vs. ≥14.75)	1.207 (0.956–1.524)	0.115	-	-
MLR (<0.345 vs. ≥0.345)	0.717 (0.576–0.892)	0.003	-	-
BLR (<0.00675 vs. ≥0.00675)	0.704 (0.56–0.885)	0.003	0.719 (0.568-0.912)	0.006
PLR (<168.745 vs. ≥168.745)	0.806 (0.64–1.014)	0.065	-	-
GLR (<38.365 vs. ≥38.365)	1.44 (0.884–2.344)	0.143	-	-
FIB-4 score (<1.285 vs. ≥1.285)	0.901 (0.732–1.11)	0.328	-	-
GAPI (<0.123 vs. ≥0.123)	1.212 (0.967–1.518)	0.095	-	-
APRI (<0.072 vs. ≥0.072)	1.326 (1.069–1.646)	0.010	-	-

*LUAD, lung adenocarcinoma; LUSC, lung squamous cell carcinoma; ANRI, aspartate transaminase-to-neutrophil ratio index; NLR, neutrophil-to-lymphocyte ratio; AGR, albumin-to-globulin ratio; SII, systemic immune inflammation index; SIRI, systemic inflammation response index; NPS, neutrophil-platelet score; F-NLR, fibrinogen-NLR score; SIS, systemic inflammation score; AFR, albumin-to-fibrinogen ratio; PNI, prognostic nutrition index; ALRI, aspartate transferase (AST)-to-lymphocyte ratio; MLR, monocyte-to-lymphocyte ratio; BLR, basophil-to-lymphocyte ratio; PLR, platelet-to-lymphocyte ratio; GLR, glutamyl transpeptidase (GGT)-to-lymphocyte ratio; FIB-4, fibrosis index based on four factors; GAPI, glutamyl transpeptidase (GGT)-to-platelet ratio; APRI, aspartate aminotransferase-to-platelet ratio index*.

As for inflammatory and nutrient biomarkers, fifteen variables including ANRI (*p* < 0.001), NLR (*p* = 0.001), AGR (*p* = 0.001), SII (*p* = 0.013), SIRI (*p* < 0.001), NPS (*p* = 0.085), F-NLR (*p* = 0.009), SIS (*p* = 0.002), AFR (*p* < 0.001), PNI (*p* = 0.003), MLR (*p* = 0.003), BLR (*p* = 0.003), PLR (*p* = 0.065), GAPI (*p* = 0.095) and APRI (*p* = 0.010) were significantly (*p* < 0.100) associated with OS in univariate Cox analysis (Table 2). While in the univariate analysis of PFS, we found that SIS (*p* = 0.142) and NPS (*p* = 0.570) were not significantly associated with tumor progression (Supplementary Table 1). As can be seen from the Figure 2C, these systemic biomarkers contained ten blood cell/serum indices (AST, N, L, Alb, Glo, PLT, M, Fib, B, GGT).

### Comparison and Determination of the Optimal Prognostic Predictive Model

**Figure 3:**
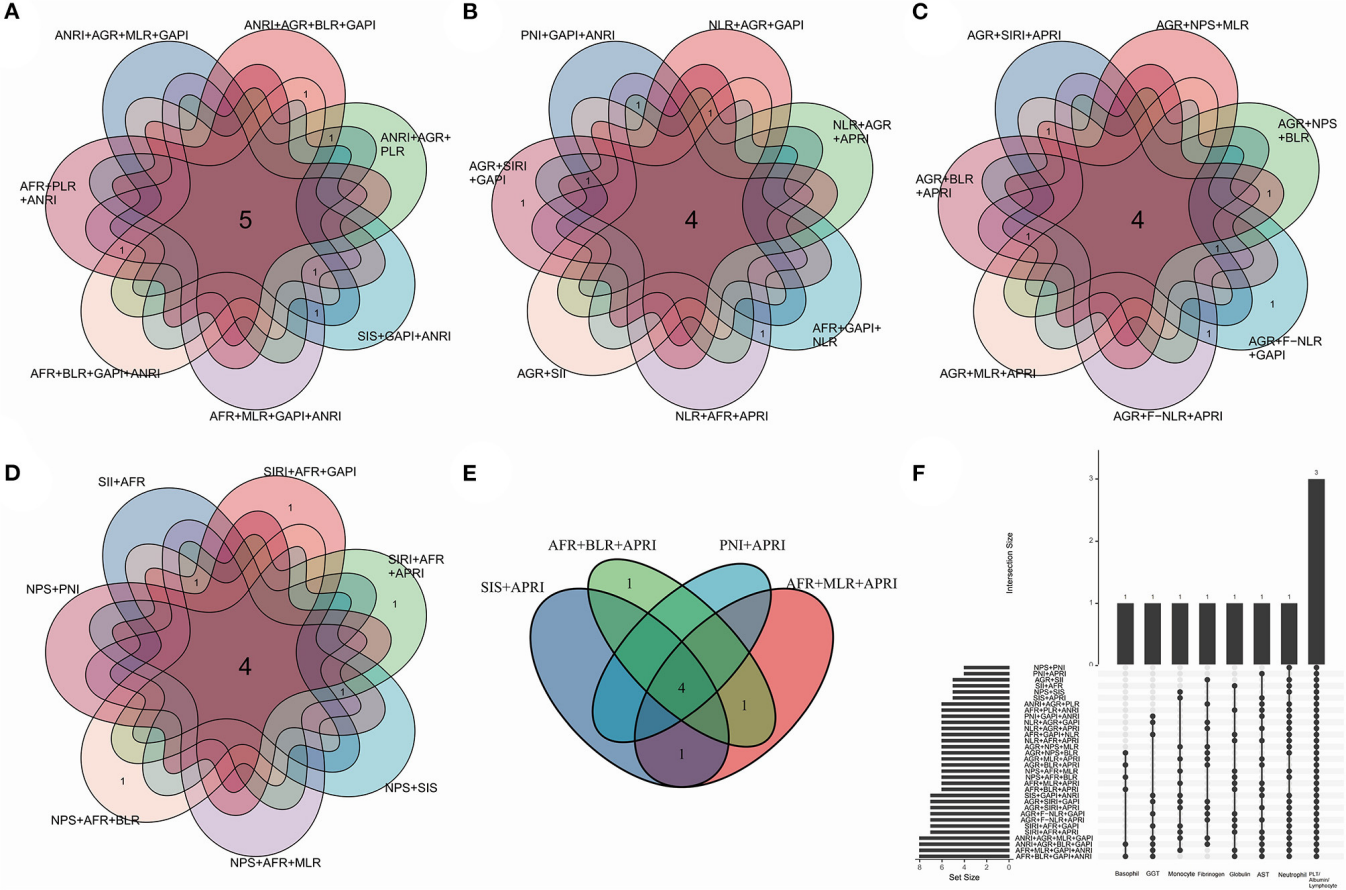
Venn plots indicating a total of thirty-two combinations of thirteen biomarkers. Demonstration in separate **(A–E)** and total **(F)** manners. Each combination contained fixed variables including gender, age, primary site, grade, stage, scope of surgery, smoking index, adjuvant therapy, surgical method (video-assisted thoracoscopic surgery or not), together with the alternative biomarkers which contains unique blood cell/serum indices. The biomarkers were significant (*p* < 0.100) in univariate Cox analysis. The numbers in the overlapping region showed the common blood cell/serum indices between two combinations.

Together with gender, age, primary site, grade, stage, scope of surgery, smoking index, VATS, adjuvant therapy and two-three alternative biomarkers (see below), a total of thirty-two combinations which their biomarkers containing non-repeating blood cell/serum indices were enumerated as candidates. They were nine fixed variables together with (1) ANRI + AGR + MLR + GAPI, (2) ANRI + AGR + BLR + GAPI, (3) ANRI+ AGR + PLR, (4) ANRI + SIS + GAPI, (5) ANRI + AFR + MLR + GAPI, (6) ANRI +AFR+BLR+GAPI, (7) ANRI + AFR + PLR, (8) ANRI + PNI + GAPI, (9) NLR + AGR + GAPI, (10) NLR + AGR + APRI, (11) NLR+AFR+GAPI, (12) NLR + AFR + APRI, (13) AGR+SII, (14) AGR + SIRI + GAPI, (15) AGR + SIRI + APRI, (16) AGR + NPS + MLR, (17) AGR + NPS + BLR, (18) AGR + F-NLR + GAPI, (19) AGR + F-NLR + APRI, (20) AGR + MLR + APRI, (21) AGR + BLR + APRI, (22) SII + AFR, (23) SIRI + AFR + GAPI, (24) SIRI + AFR + APRI, (25) NPS + SIS, (26) NPS + AFR + MLR, (27) NPS + AFR + BLR, (28) NPS+PNI, (29) SIS + APRI, (30) AFR + MLR + APRI, (31) AFR + BLR + APRI and (32) PNI + APRI (Figure 3). All of them were subjected to multivariate Cox analyses. Consequently, three prognostic models in which the biomarkers and non-biomarkers could coexist stood out with significance (*p* < 0.05), including grade, age and stage together with (1) ANRI, (2) BLR, (3) BLR + AGR. Other models did not contain variables of biomarker.

**Figure 4:**
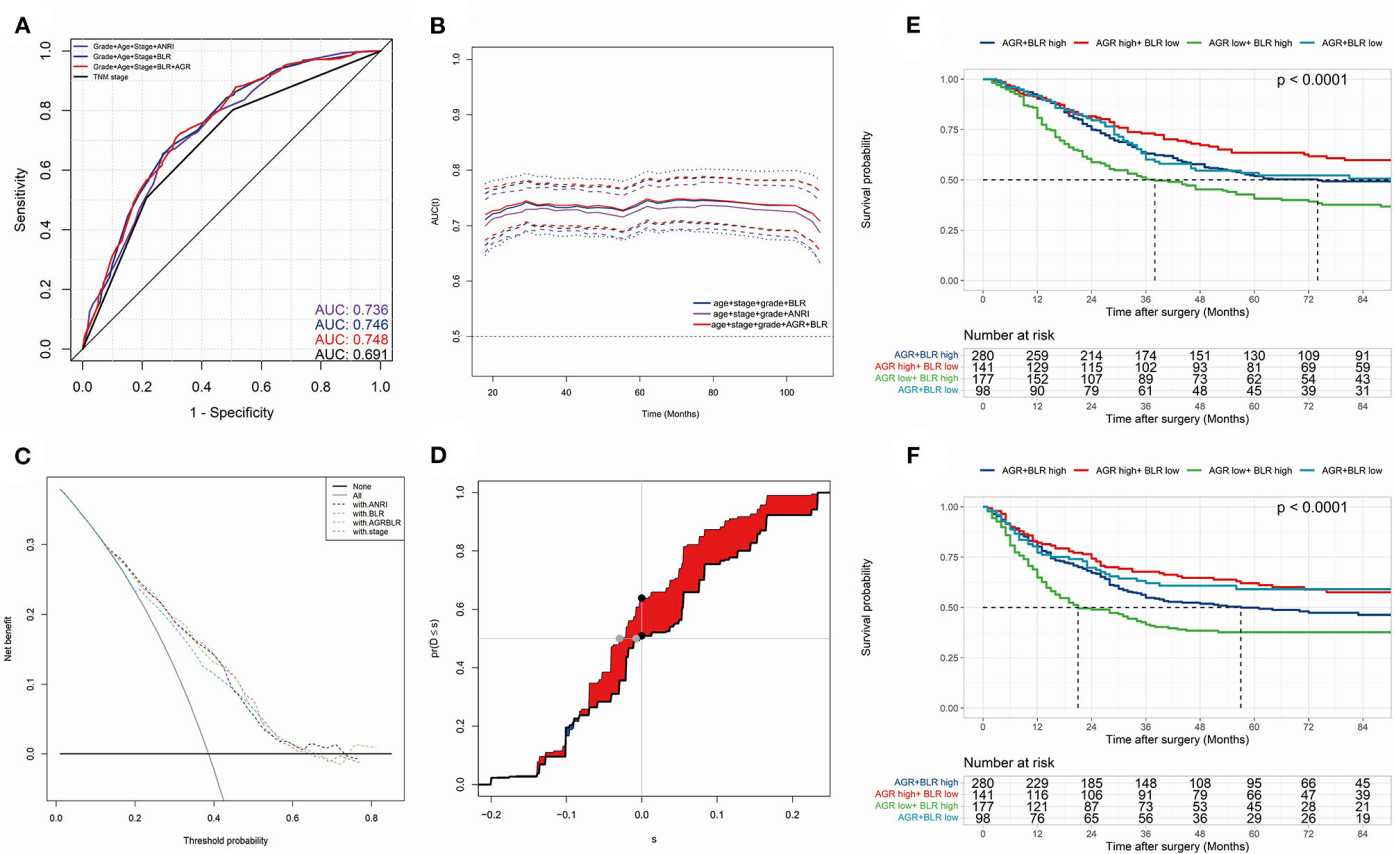
**(A)** the receiver operating characteristic (ROC) curves in OS of prognostic models; **(B)** time-dependent area under curve (AUC) in OS of the prognostic models. **(A,B)** were calculated by the total points of each variable in different Cox models. **(C)** time-dependent (3-year) decision curve analysis (DCA) in OS of the prognostic models, of which the variables were significant (*p* < 0.050) in multivariate Cox analysis. **(D)** concretization of Integrated Discrimination Improvement (IDI) for comparing the performance of prognostic models (containing variables grade, stage, age, BLR and AGR) and TNM stage. **(E,F)** Kaplan-Meier analyses and log-rank tests of AGR together with BLR in OS **(E)** and PFS **(F)** in the training group.

The model with biomarkers BLR + AGR stood out for its better performance. The ROC curves (Figure 4A) and time-dependent AUC (Figure 4B) showed its highest AUC value in the training group for OS among the three models. Although time-dependent (3-year) DCA (Figure 4C) indicated their similar clinical utility, the model with BLR+AGR indicated the highest IDI (Figure 4D, 0.035). The IDI of model with ANRI and BLR were 0.029 and 0.031, respectively. Besides, the C-index of the model with BLR + AGR was 0.690, higher than the model with ANRI (0.680) and BLR (0.687). To evaluate its stratified performance for excessive inflammatory or nutrition-deficient status, we performed Kaplan-Meier analyses of the two biomarker combinations (Figures 4E,F). The pairwise log-rank tests of each combination showed that patients with higher BLR and lower AGR had the significantly worse prognosis. In summary, we finally chose the model grade+age+stage+BLR+AGR as the optimal prognostic model, and its subsequent validation was conducted.

### Model Validation: Predictive Accuracy and Clinical Utility

**Figure 5:**
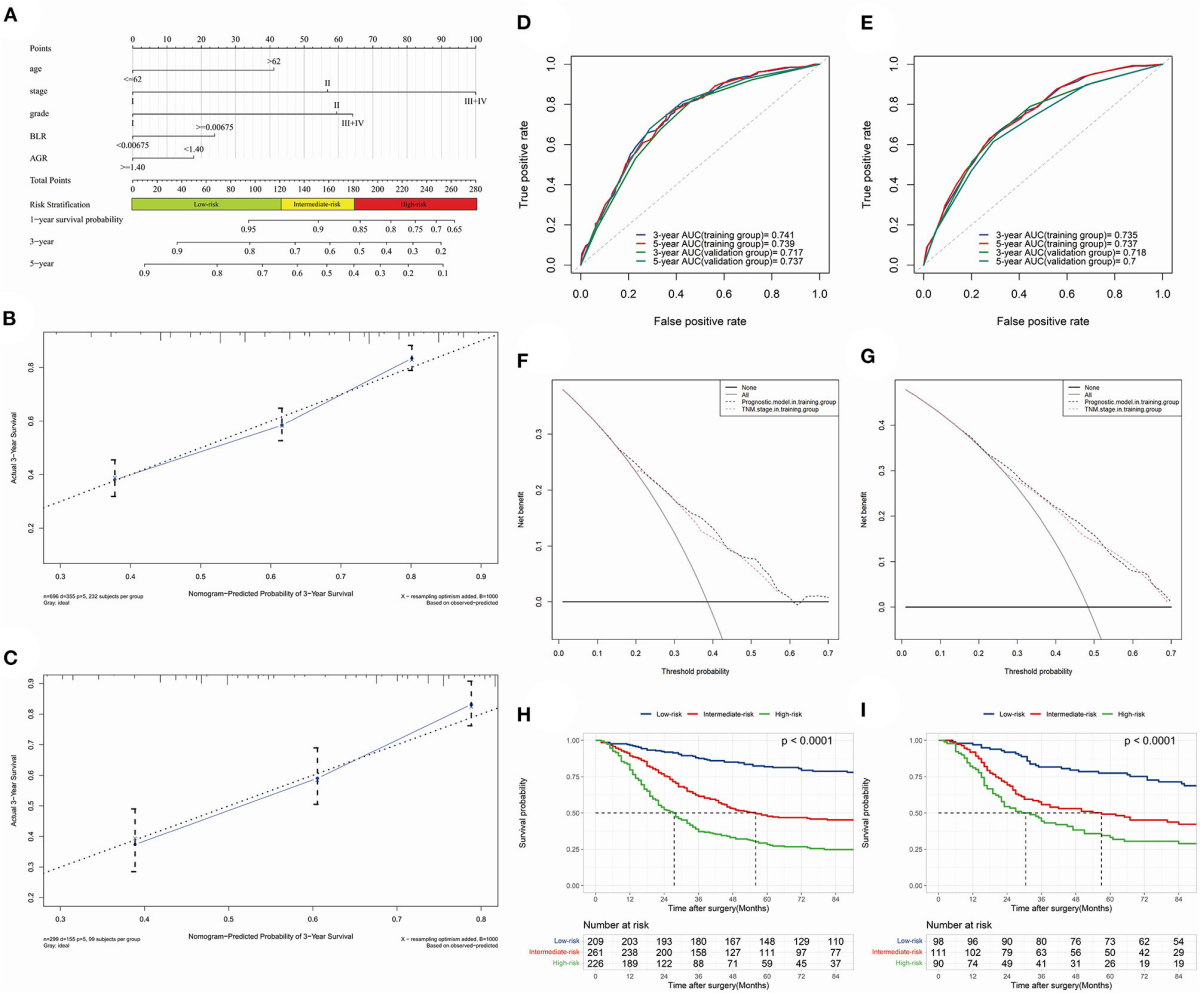
The optimal prognostic model constructed by training group and time-dependent validations. **(A)** nomogram predicting 1-, 3- and 5-year survival probability with risk stratification; **(B)** (training group) and **(C)** (validation group), 3-year calibration plot; **(D)** (OS) and **(E)** (PFS), 3- and 5-year receiver operating characteristic (ROC) curves and area under curves (AUC); **(F)** (3-year) and **(G)** (5-year), time-dependent decision curve analysis (DCA); **(H)** (training group) and **(I)** (validation group), Kaplan-Meier analysis and log-rank test indicating prognostic stratification of high-, intermediate- and low-risk.

At last, a prognostic nomogram with risk stratification containing 1-, 3- and 5-year survival probability was conducted (Figure 5A). The calibration of the model could be reflected by the closeness of the data points to the diagonal line. Time-dependent calibration plots of 3-year (Figures 5B,C) and 5-year (Supplementary Figures 3A,B) survival showed a good performance both in the training and validation group. To validate the discrimination of the model, we performed time-dependent ROC for 3-year and 5-year survival in the training (Figure 5D) and validation (Figure 5E) group. The 3- and 5-year AUC of OS in the training group were 0.741 and 0.739; while the 3- and 5-year AUC of OS in the validation group were 0.717 and 0.737, respectively. The nomogram demonstrated an accurate prediction for 3- and 5-year OS of NSCLC patients.

C-index in training and validation group were 0.690 and 0.683, respectively. The clinical utility of the model was investigated by time-dependent DCA curves compared with the TNM stage in the two groups (Figures 5F,G, 3-year DCA; Supplementary Figures 3C,D, 5-year DCA). Risk stratification divided patients into low-risk (≤ 120.99 points), intermediate-risk (121.90–181.39 points) and high-risk (≥182.00 points) groups. Kaplan-Meier analysis and log-rank test demonstrated its favorable performance of stratification in training (*p* < 0.001) and validation (*p* < 0.001) groups (Figures 5H,I). What's more, the 3- and 5-year NRI in training group were 0.129 (0.072–0.246, *p* < 0.001) vs. 0.125 (0.055–0.232, *p* < 0.001), respectively.

What's more, we broke down the chosen inflammatory biomarkers (BLR and AGR) into basophil, lymphocyte, albumin and globulin, then included them into univariate and multivariate Cox analysis in the training group (Supplementary Table 2). The four blood cell or serum indices were all significant (*p* < 0.100) in the univariate Cox analysis, however, they were not significant (*p* < 0.050) with variables age + stage + grade in the multivariate Cox analysis. Then we compared the AUC and C-index of the models (age + stage + grade + BLR + AGR, age + stage + grade, TNM stage) in the training and validation group (Supplementary Figure 4). The C-index were 0.650 (TNM stage) vs. 0.676 (age + stage + grade) vs. 0.690 (age + stage + grade + BLR + AGR) in the training group, and 0.663 (TNM stage) vs. 0.679 (age + stage + grade) vs. 0.683 (age + stage + grade + BLR + AGR) in the validation group, respectively. The results indicated that the prognostic model with inflammatory biomarkers had better prognostic performance than the models with clinicopathological variables only.

### Model Validation: Collinearity and Fitness

**Figure 6:**
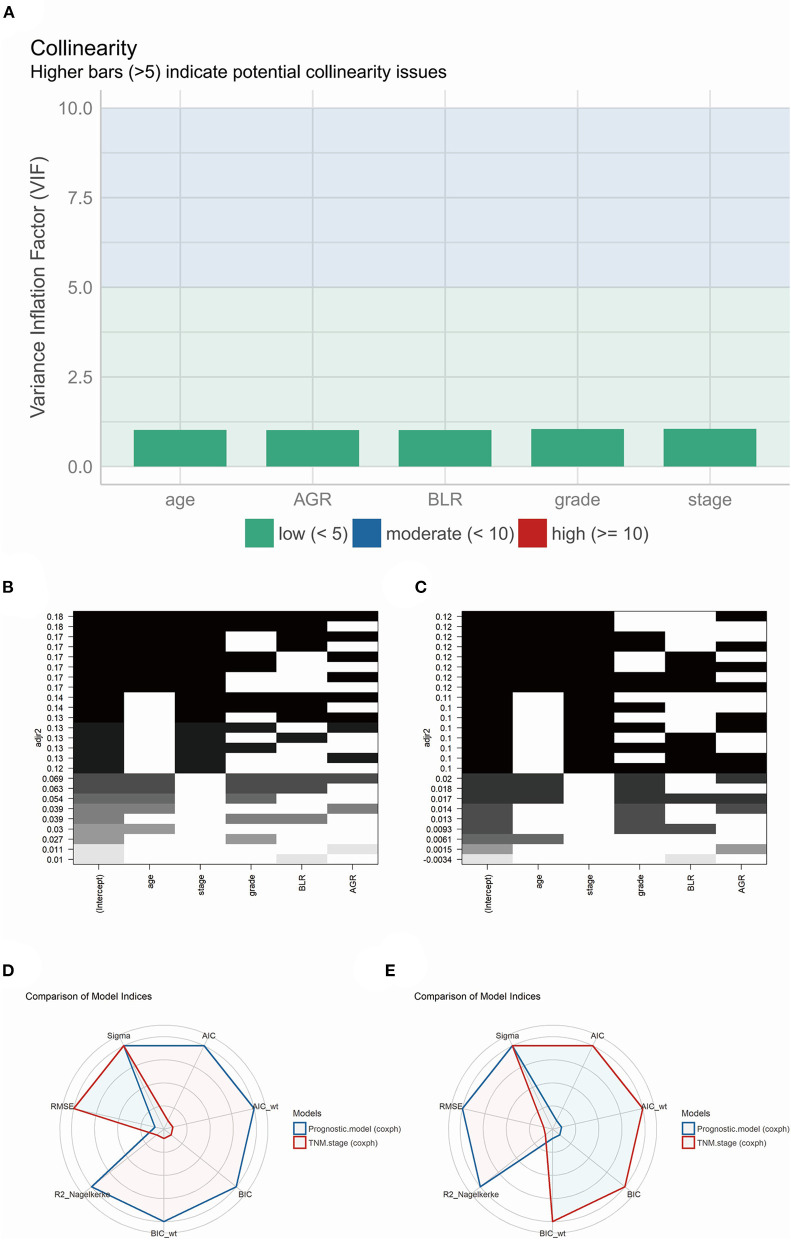
**(A)** The model performance of collinearity. High bars (>5) indicate potential collinearity issues. **(B)** (training group) and **(C)** (validation group), the model performance of all-subsets regression analysis. **(D)** (training group) and **(E)** (validation group), radar plot indicating the comparison of different model indices between TNM staging system and prognostic model.

The performance of the model's collinearity was also tested. The low variance inflation factors (VIF) of the variables indicated weak collinearity issues of the model (Figure 6A). All-subsets regression analysis revealed that the highest adjusted R-square (training group, 0.180; validation group, 0.120) could be reached when all the five variables were included (Figures 6B,C). Besides, Akaike information criterion (AIC), Bayesian information criterion (BIC) and root mean squared error (RMSE) were also calculated and visualized compared with model of TNM stage in training (Figure 6D) and validation (Figure 6E) group. In the training group, the AIC (4260.376) and BIC (4279.737) of the prognostic model were less than that of the TNM stage model (AIC, 4294.740; BIC, 4298.612), indicating an acceptable performance for the model's fitness.

On the other hand, the RMSE of the model (0.731) was higher than the TNM stage (0.723) in the training group, which showed a slightly larger deviation between the observed and true outcome of patients compared to the TNM stage model (Figure 6D). Interestingly, the result in the validation group was converted yet with little difference between the prognostic model and TNM stage model. The prognostic model revealed a higher AIC (1613.084) and BIC (1628.301) than the TNM stage (AIC, 1612.919; BIC, 1615.962), and a equal RMSE (prognostic model and TNM stage, 0.739) (Figure 6E).

### Subgroup Analysis

**Figure 7:**
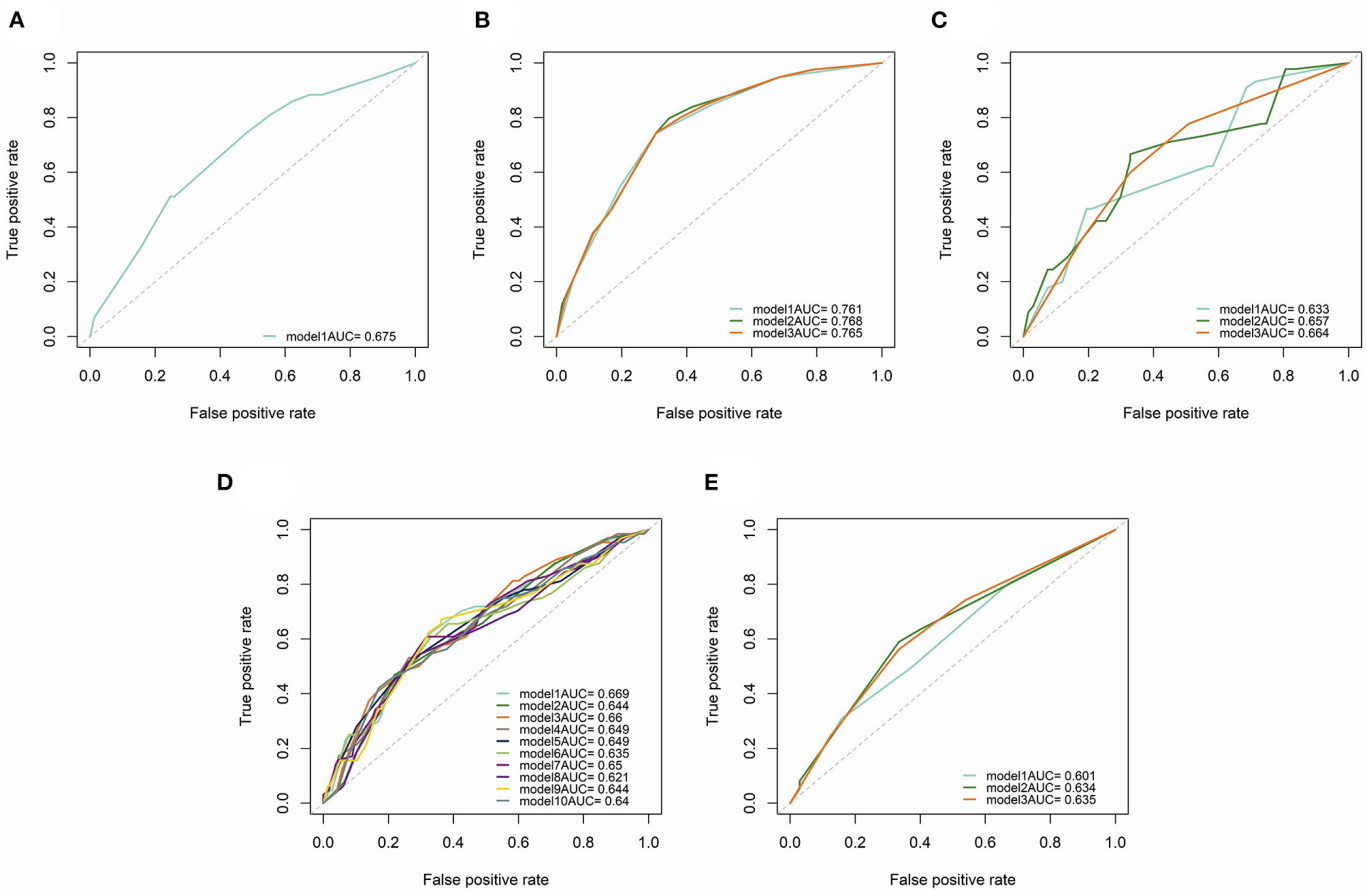
Time-dependent (3-year) ROC curves and AUC of different prognostic models in subgroup analysis. **(A)** patients underwent sublobectomy. **(B)** patients underwent lobectomy. **(C)** patients underwent extended lobectomy. **(D)** patients in stage I. **(E)**, patients in stage II.

Subgroup analysis demonstrated the possible collocations and prognostic performance of all models in different surgical scopes and TNM stages (Table 3 and Figure 7). The prognostic efficacy of models were quite different, and the variables were also varied. However, in the models with the high prognostic power, the inflammatory biomarkers were still AGR (C-index = 0.715, 3-year AUC = 0.768), BLR (C-index = 0.713, 3-year AUC = 0.660) and ANRI (C-index = 0.712, 3-year AUC = 0.765), which showed their relatively high prognostic correlation with NSCLC patients.

**Table 3:** Subgroup analyses of prognostic models for surgical scope and TNM stage.

	**Subgroups**	**Number of possible**	**Models which significant in multivariate**	**C-index**
		**combinations**	**cox analysis**	
Surgical scope	Sublobectomy	3	Grade + primary site + VATS + pyrexia after surgery	0.703
	Lobectomy	16	Model 1: age + stage	0.706
			Model 2: age + stage + AGR	0.715
			Model 3: age + stage + ANRI	0.712
	Extended lobectomy	5	Model 1: age + primary site	0.691
			Model 2: age + primary site + APRI	0.711
			Model 3: primary site + stage + AFR	0.703
	Pneumonectomy	3	-	-
TNM stage	I	16	Model 1: grade + age + surgical scope + laterality + primary site + NLR	0.710
			Model 2: grade + age + surgical scope + laterality + primary site	0.700
			Model 3: grade + age + surgical scope + laterality + primary site + BLR	0.713
			Model 4: grade + age + surgical scope + laterality + primary site + PLR	0.712
			Model 5: grade+surgical scope+FIB-4+NLR	0.668
			Model 6: grade + surgical scope + laterality + primary site + FIB-4 + SIRI	0.687
			Model 7: grade + surgical scope + laterality + primary site + FIB-4 + F-NLR	0.684
			Model 8: grade + surgical scope + laterality + primary site + FIB-4	0.677
			Model 9: grade + surgical scope + laterality + primary site + FIB-4 + MLR	0.686
			Model 10: grade + surgical scope + laterality + primary site + FIB-4 + BLR	0.692
	II	3	Model 1: primary site+FIB-4+pyrexia after surgery	0.588
			Model 2: primary site + age + NPS	0.624
			Model 3: primary site+ age + APRI	0.636
	III + IV	4	-	-

To investigate the prognostic relationship between different surgical scopes and inflammatory biomarkers, Kaplan-Meier analyses and log-rank tests in subgroups (high- and low-level groups of BLR and AGR) were performed (Supplementary Figure 5). Interestingly, regardless of BLR and AGR (or OS and PFS), the log-rank tests of high-level subgroups were all significant (*p* < 0.001), indicating that lobectomy is the preferred surgical scope compared with the others. As a contrast, no variances (*p* > 0.050) were observed between each surgical scopes in low-level subgroups, except for AGR subgroup (Supplementary Figure 5F, PFS, *p* = 0.025).

## Discussion

When the process of inflammation is activated, acute inflammatory agents act on the human body and participate pivotally in infection resistance and wound healing (23). However, continuous exposure to exogenous or endogenous inflammatory stimulus could lead to chronic inflammation, which could be permanently detrimental to tissues, such in the case of chronic inflammatory illness such as chronic obstructive pulmonary disease (COPD) and diabetes (24–26). Worse still, the constant stimulus by chronic inflammatory factors could result in severe parenchymal cell degeneration, necrosis, and metabolic dysfunction, ultimately leading to carcinogenesis (27). Such phenomenon of inflammation-elicited tumorigenesis can be verified by the analysis of pathological sections, which show that solid tumor cells are infiltrated and surrounded by multiple immune cells (innate and adaptive) (28). Consequently, inflammation can be regarded as a central hallmark of cancer for its tumor-enabling capacity (15).

Interactions between pro-tumorigenic inflammation (including local immune response and systemic inflammation) and carcinoma are intricate (13, 29). The local immune response, which could tremendously impact constituents of the TME toward a more tumor-permissive state and block anti-tumor immunity, is widely confirmed to promote tumorigenesis in almost every aspect (30). Under the influence of pro-tumorigenic inflammation, cancer could also be facilitated by receiving tumor-promoting signals (30). A previous study revealed that oxidative stress induced by inflammatory stimuli could lead to recurrent genomic rearrangements, thus leading to prostate cancer development (31). Another research concerning the whole-genome sequencing of 149 NSCLC cases in China showed that the accumulation of EGFR (Epidermal Growth Factor Receptor) mutations may be ascribed to inflammatory infiltration, especially for never-smokers (32). Conversely, secretomes originating from tumors were identified in the systemic circulation of patients with carcinoma and could regulate distant organs, including bone marrow, liver, and spleen (33). Cytokines, small inflammatory proteins, and immune cells thus accumulate in the TME and systemically, accompanied by relevant clinical manifestations, which could be quantified by preoperative examinations including blood biochemical, blood routine test, temperature chart, etc. (34). Therein, the level of systemic inflammatory biomarkers for patients undergoing surgical treatment gained great interest (35–37).

Nutrients can influence tumor progression by affecting multiple cellular processes, such as regulating cell growth and death and upregulating the expression of oncogenes. Previous studies had demonstrated that many nutrients, including calcium, fiber, and vitamin D, have strong relevance to tumor metabolism and drug efficacy (16). For example, other than the role of glucose in supplying tumor proliferation with energy, its consumption also influences tumorigenesis by promoting the secretion of insulin, which is an oncogenic signaling factor (17). Another finding is that in a mouse model of leukemia, the response to methotrexate (MTX) was reinforced by adding histidine to the diet of the mice (38). Moreover, the interaction between nutrients might also participate in cancer development. Free fatty acids can facilitate tumor growth, while their effect can be blocked by ionized calcium in the colonic lumen (39). Therefore, it's worthwhile investigating an antitumor strategy termed ‘tumor starvation': it is based on the principle to suppress tumor growth by restricting the supply of nutrients which are essential for some tumors, e.g., glutamine, serine, and folate (40). However, dietary guidance aiming at cancer treatment has not yet drawn much attention, and there is no specific guideline for clinical practice. We hope to inspire others not only to focus on the prognosis of NSCLC correlated to nutrient biomarkers, but also to combine cancer treatment, or even prevention, with dietary modifications.

In our study, we concluded that AGR, BLR and ANRI could together predict the prognosis of patients with NSCLC. AGR composes of serum albumin and globulin, which represent the nutritional status and level of inflammatory activity, respectively. For patients with advanced cancer, the albumin decrease because of its consumption due to cancer cachexia (41). Moreover, there is evidence that albumin could be indicative of inflammatory activity (42). Globulin, e.g., immunoglobulin (Ig) and chemokines, mediates antigen resistance and enhances the induction of immune cells on solid tumors. The prognostic value of preoperative AGR has been studied by several researchers. Some studies concluded that patients with a relatively low level of AGR had a better prognosis in NSCLC (43). However, the present study drew a different conclusion, indicating that patients whose AGR ≥1.375 had a better survival than those not, similarly to previous research (44). Aspartate aminotransferase (AST) ubiquitously exists mainly in cardiomyocytes and hepatocytes, participating in amino acid metabolism and tricarboxylic acid cycles. Its serum concentration rises due to increased permeability of the cell membrane, which is an important indicator reflecting the inflammatory state (45, 46).

Another two biomarkers related to cancer are BLR and ANRI. As the lowest content in peripheral leukocytes, basophils are widely thought to play an important role in allergic diseases. In addition, basophils can also promote tumor progression by secreting cytokines and chemokines, such as interleukin (IL)-4 and IL-13 (47). De Monte et al. found that IL-4 derived from basophils could promote polarizing the M2 macrophage, a subtype that enhancing tumor angiogenesis and metastasis, then play an indirect role in promoting cancer (48). Another evidence supporting the cancer-promoting phenotype of basophils is that, when basophils and lung cancer cell line A549 were cocultured together, the former could produce IL-13, which could in turn promote the proliferation and migration of the latter (49, 50). Interestingly, some researchers revealed that lung resident basophils, which located near the alveoli and exhibiting a lung-specific phenotype, are very different from basophils in the peripheral circulation (51). ANRI, which combines the AST with neutrophils count, is involved in the prognostic or diagnostic prediction for hepatocellular carcinoma and intrahepatic cholangiocarcinoma (ICC) (52–54). Liu et al. evaluated the prognostic performance of the preoperative ANRI in ICC after surgery and concluded that a lower ANRI (≤ 6.7) was an independent predictor for dismal prognosis (54). Nevertheless, few studies are substantiating the prognostic value of ANRI in NSCLC. Consistently with Liu et al. study in ICC, we observed that a lower (<4.91) level of pretreatment ANRI was significantly associated with a poor prognosis for NSCLC patients.

Our study incorporated multiple systemic inflammatory biomarkers, which derived from blood cell/serum indices of preoperative blood biochemical and blood routine tests. These indices are non-invasive and could be easily assessed in clinical practice. Besides, we also collected the consecutive body temperature of patients, which also reflects the overall inflammatory status before surgery. These results might guide the clinicians to determine the appropriate time for conducting surgical procedures and anti-inflammatory treatment during the perioperative period. Moreover, we conducted operating characteristic (ROC) curves for each biomarker to verify their prognostic performance, as well as to identify the optimal cutoff value of each biomarker. The continuous variables of inflammation were then transformed into high- and low-level groups for subsequent univariate and multivariate Cox analysis. Finally, we constructed a nomogram for survival prediction of patients with NSCLC after surgical treatment, which contained variables including age, grade of tumor differentiation, TNM stage, BLR and AGR. Compared with other researches of a prognostic model concerning inflammatory biomarkers, we established the risk stratification for each individual due to the total points, and satisfactory significance of stratification was confirmed both for OS and PFS. The verification of the model's collinearity and fitness also demonstrated good performance and promising clinical utility.

There were a few limitations in our study. First, this study was a single-center retrospective analysis, and selection bias was present due to the lack of external validation from another constitution. Next, several indices which reflect the inflammatory status of NSCLC patients were not involved, such as CAR (containing CRP), ALI (containing BMI), LIPI (lung immune prognostic index, containing LDH [lactate dehydrogenase]). Thirdly, other inflammatory diseases could interfere with the level of inflammatory biomarkers, e.g., type 2 diabetes and autoimmune diseases. Finally, as a result of the progress of surgical methods and other adjuvant treatments, the prognosis of patients may be affected compared to that from 10 to 15 years ago. In conclusion, a multi-center and large-population study involving more comprehensive inflammatory biomarkers is required for further validation of our findings.

## Data Availability Statement

The raw data supporting the conclusions of this article will be made available by the authors, without undue reservation.

## Ethics Statement

The studies involving human participants were reviewed and approved by Biomedical Research Ethic Committee of Shandong Provincial Hospital. The patients/participants provided their written informed consent to participate in this study.

## Author Contributions

KW, QZ, and JD: conception and design. JD and GW: administrative support. KW, TY, and JD: provision of study materials or patients. QZ, TY, and DG: collection and assembly of data. QZ and JL: data analysis and interpretation. All authors: manuscript writing and final approval of manuscript.

## Funding

This work was supported by the National Natural Science Foundation of China [82102700]; Natural Science Foundation of Shandong Province [ZR2019PH002]; Clinical Medicine Science and Technology Innovation Plan of Jinan City [202019058]. The funders had no role in study design, data collection and analysis, decision to publish or preparation of the manuscript.

## Conflict of Interest

The authors declare that the research was conducted in the absence of any commercial or financial relationships that could be construed as a potential conflict of interest.

## Publisher's Note

All claims expressed in this article are solely those of the authors and do not necessarily represent those of their affiliated organizations, or those of the publisher, the editors and the reviewers. Any product that may be evaluated in this article, or claim that may be made by its manufacturer, is not guaranteed or endorsed by the publisher.

## Supplementary Material

Supplementary Figure 1 The overall survival **(A)** and progression-free survival **(B)** of total patients, patients in training and validation group, respectively.

Supplementary Figure 2 Kaplan-Meier analyses and log-rank tests of pyrexia before surgery in OS **(A)** *p* = 0.530 and PFS **(B)**, *p* = 0.600, pyrexia after surgery in OS **(C)** *p* = 0.430 and PFS **(D)** *p* = 0.003, hyperpyrexia before surgery in OS **(E)** *p* = 0.200 and PFS **(F)** *p* = 0.730, hyperpyrexia after surgery in OS **(G)** *p* = 0.240 and PFS **(H)** *p* = 0.900 in training group, respectively.

Supplementary Figure 3 Time-dependent (5-year) calibration plots in training group **(A)** and validation group **(B)**. Time-dependent decision curve analysis (DCA) curves in training group. **(C)**, 3-year; **(D)**, 5-year.

Supplementary Figure 4 Time-dependent ROC curves and AUC of different prognostic models in training and validation group. **(A)** 3-year, training group; **(B)** 5-year, training group; **(C)** 3-year, validation group; **(D)** 5-year, validation group.

Supplementary Figure 5 Kaplan-Meier analyses and log-rank tests of patients in BLR and AGR subgroup stratified by scope of surgery. **(A,B)** high-level of AGR; **(E,F)** low-level of AGR; **(C,D)** high-level of BLR; **(G,H)** low-level of BLR. **(A,C,E,G)** overall survival (OS); **(B,D,F,H)** progression-free survival (PFS).

Supplementary Table 1 Univariate and multivariate COX regression analysis of progression-free survival in training group.

Supplementary Table 2 Univariate and multivariate COX regression analysis of overall survival in training group.

## Abbreviations

NSCLC: non-small cell lung cancer
ROC: receiver operating characteristic
DCA: decision curves analysis
OS: overall survival
PFS: progression free survival
CI: confidence interval
AUC: area under the curve
C-index: concordance index
IDI: integrated discrimination improvement
AIC: Akaike information criterion
BIC: Bayesian information criterion
RMSE: Nagelkerke R-Square and root mean squared error
TME: tumor microenvironment
COPD: chronic obstructive pulmonary disease
AST: aspartate aminotransferase
ALT: alanine aminotransferase
Alb: albumin
Glo: globulin
Fib: fibrinogen
GGT: gamma-glutamyl transferase
M: monocyte
L: lymphocyte
N: neutrophil
E: eosinophil
B: basophil
PLT: platelet
NLR: neutrophil-to-lymphocyte ratio
PLR: platelet-to-lymphocyte ratio
BLR: basophil-to-lymphocyte ratio
MLR: monocyte-to-lymphocyte ratio
SIRI: systemic inflammation response index
SII: systemic immune inflammation index
AGR: albumin-to-globulin ratio
AFR: albumin-to-fibrinogen ratio
PNI: prognostic nutritional index
GAPI: glutamyl transpeptidase-to-platelet ratio
GLR: GGT-to-lymphocyte ratio
ALRI: aspartate transferase-to-lymphocyte ratio
ANRI, aspartate transferase: AST-to-neutrophil ratio
APRI: AST-to-platelet ratio
FIB-4 score: fibrosis index based on four factors
SIS: systemic inflammation score
NPS: neutrophil-platelet score
F-NLR: fibrinogen-NLR score
LIPI: lung immune prognostic index.
