# Supplementary figures and images for: The Prognostic Value of Multiple Systemic Inflammatory Biomarkers in Preoperative Patients With Non-small Cell Lung Cancer
Source: Front Surg. 2022 Apr 4;9:830642. doi: 10.3389/fsurg.2022.830642 (PMC9013845; doi:10.3389/fsurg.2022.830642)

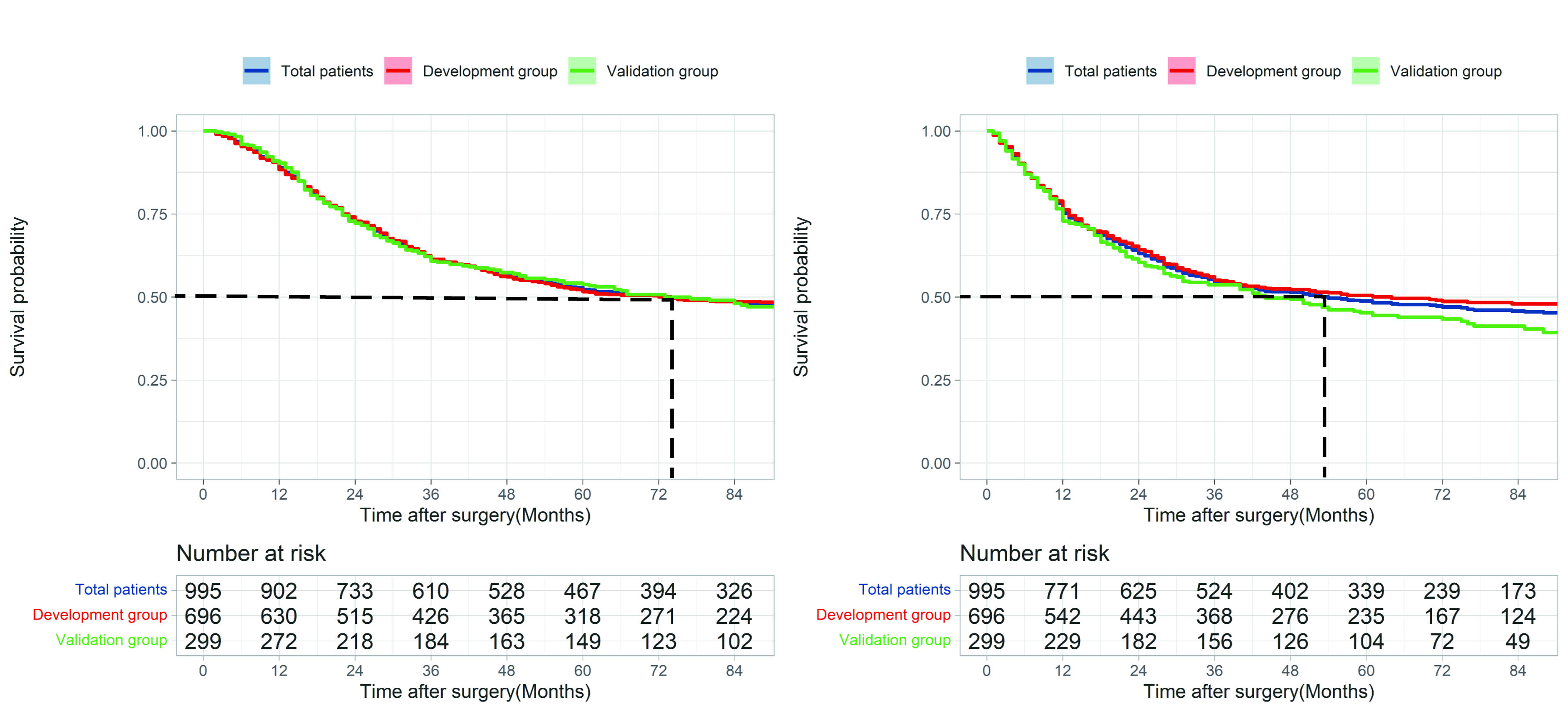

Supplement: Supplementary Figure 1 — The overall survival (A) and progression-free survival (B) of total patients, patients in training and validation group, respectively. [file Image_1.JPEG]

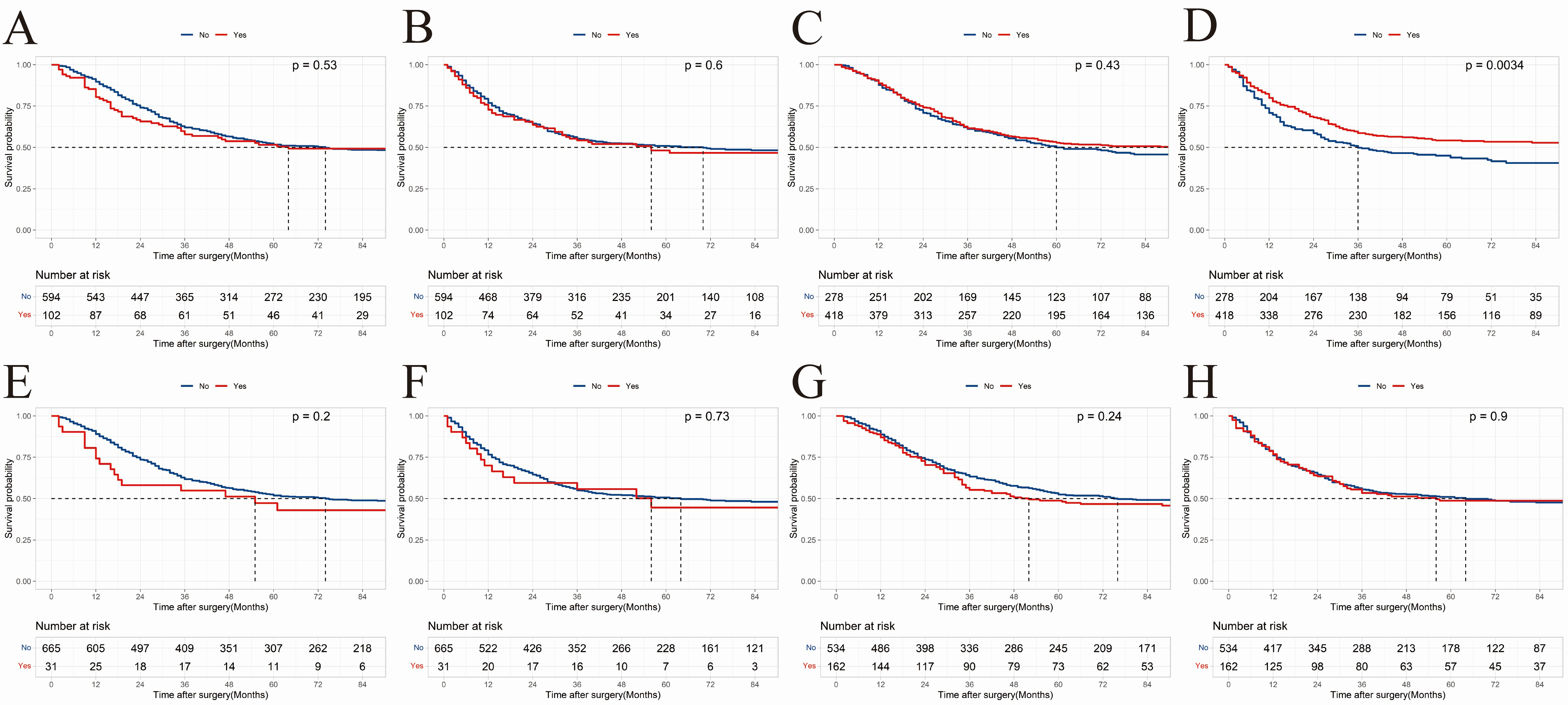

Supplement: Supplementary Figure 2 — Kaplan-Meier analyses and log-rank tests of pyrexia before surgery in OS (A) p = 0.530 and PFS (B), p = 0.600, pyrexia after surgery in OS (C) p = 0.430 and PFS (D) p = 0.003, hyperpyrexia before surgery in OS (E) p = 0.200 and PFS (F) p = 0.730, hyperpyrexia after surgery in OS (G) p = 0.240 and PFS (H) p = 0.900 in training group, respectively. [file Image_2.JPEG]

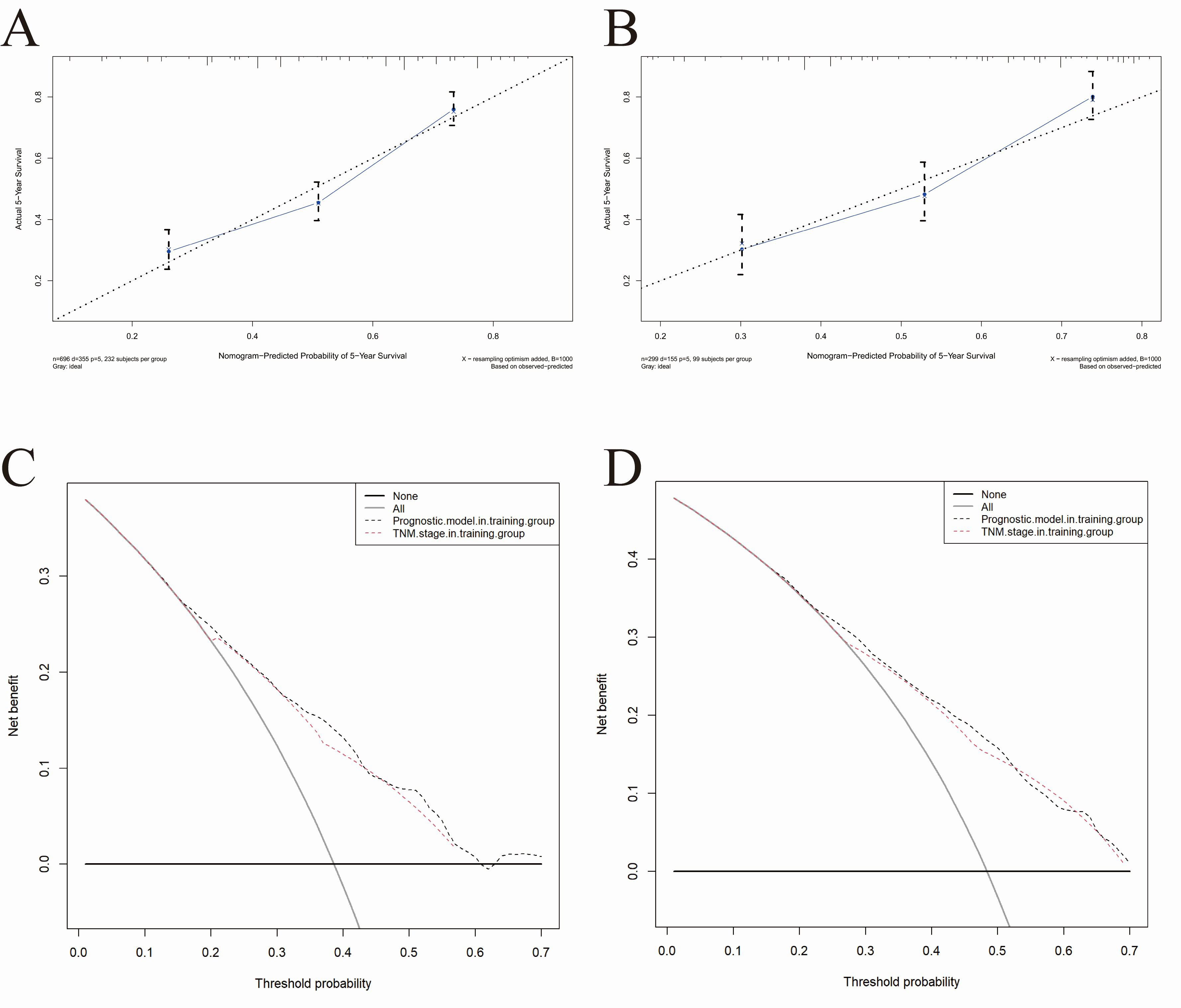

Supplement: Supplementary Figure 3 — Time-dependent (5-year) calibration plots in training group (A) and validation group (B). Time-dependent decision curve analysis (DCA) curves in training group. (C), 3-year; (D), 5-year. [file Image_3.JPEG]

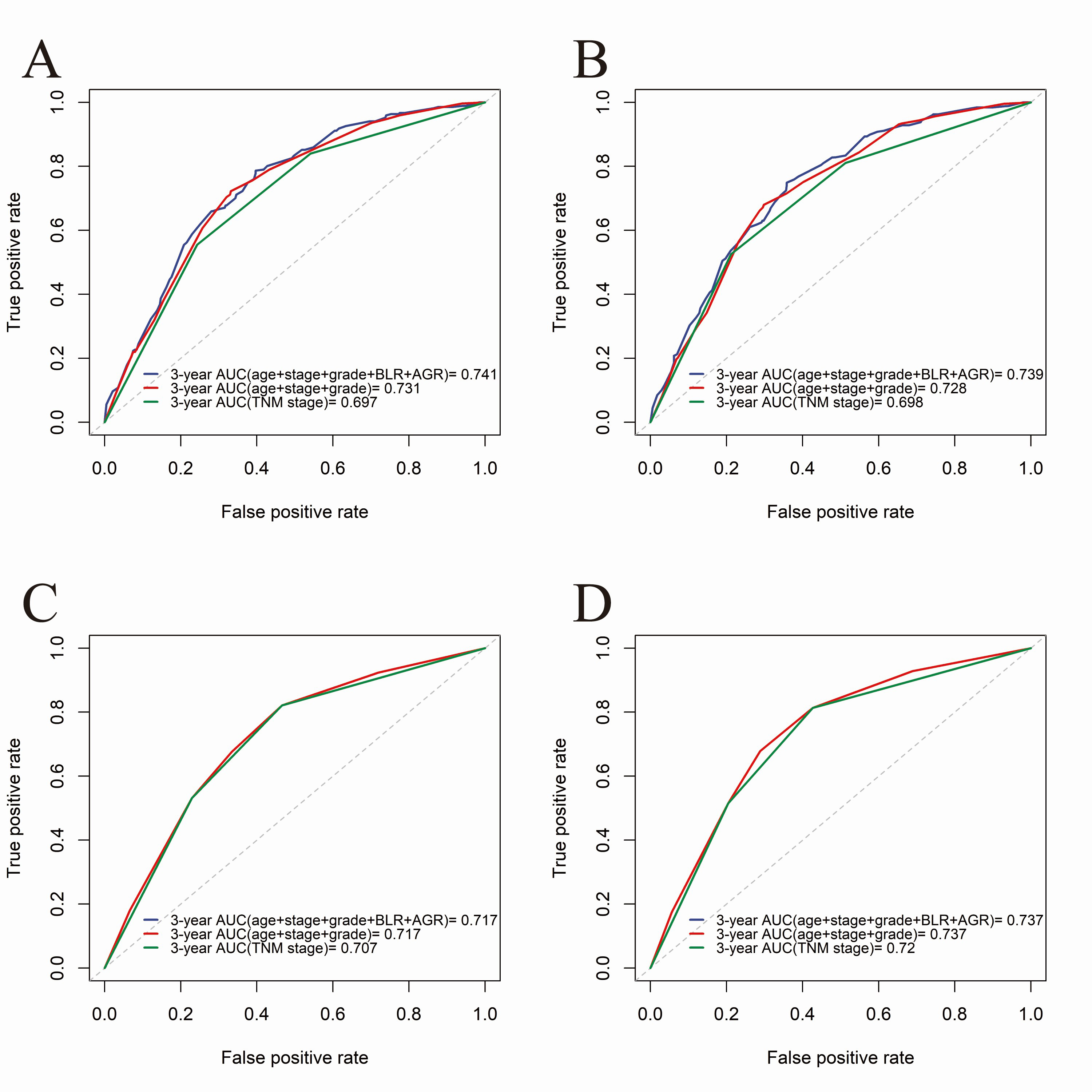

Supplement: Supplementary Figure 4 — Time-dependent ROC curves and AUC of different prognostic models in training and validation group. (A) 3-year, training group; (B) 5-year, training group; (C) 3-year, validation group; (D) 5-year, validation group. [file Image_4.JPEG]

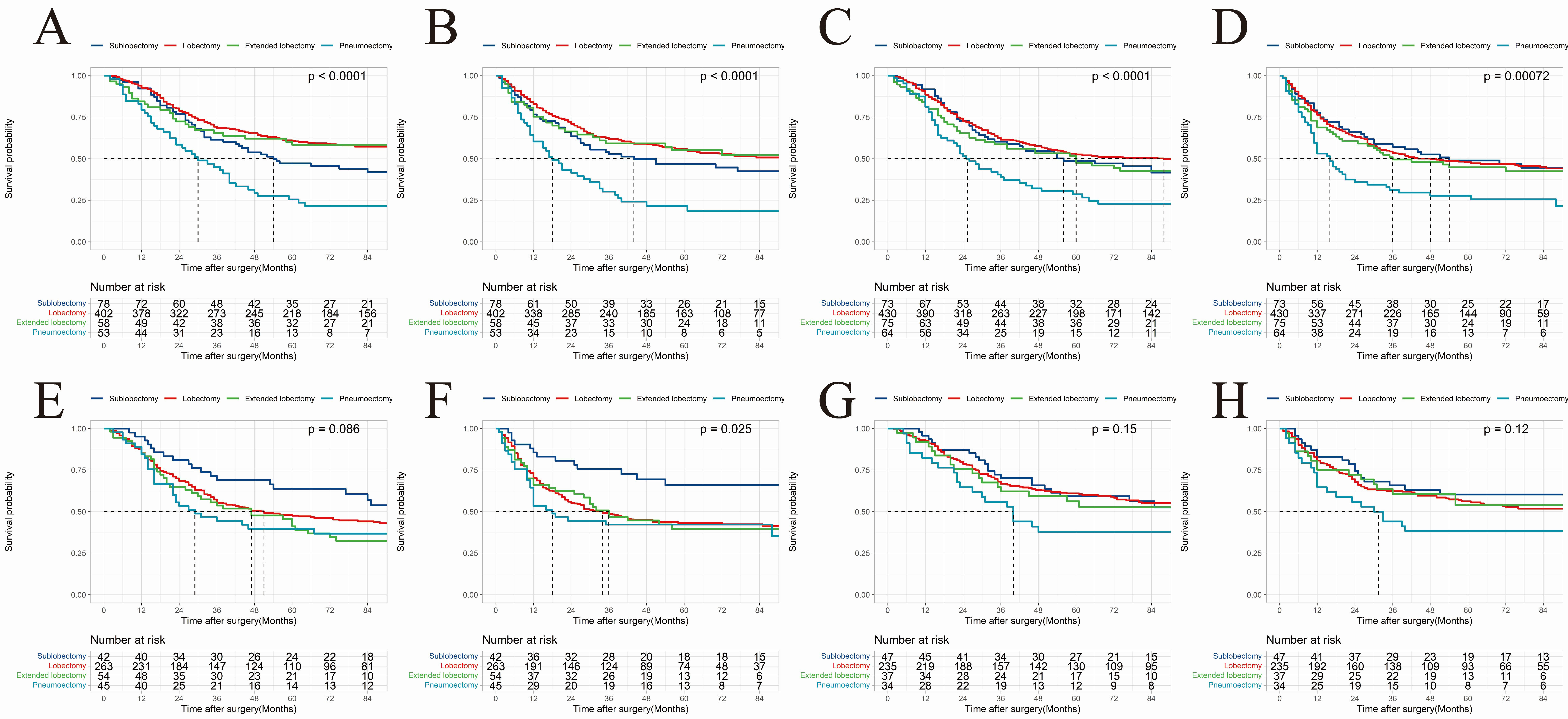

Supplement: Supplementary Figure 5 — Kaplan-Meier analyses and log-rank tests of patients in BLR and AGR subgroup stratified by scope of surgery. (A,B) high-level of AGR; (E,F) low-level of AGR; (C,D) high-level of BLR; (G,H) low-level of BLR. (A,C,E,G) overall survival (OS); (B,D,F,H) progression-free survival (PFS). [file Image_5.JPEG]
