# Supplementary material for: The Prognostic Value of Multiple Systemic Inflammatory Biomarkers in Preoperative Patients With Non-small Cell Lung Cancer
Source: Front Surg. 2022 Apr 4;9:830642. doi: 10.3389/fsurg.2022.830642 (PMC9013845; doi:10.3389/fsurg.2022.830642)
Supplement: Supplementary Table 1 — Univariate and multivariate COX regression analysis of progression-free survival in training group. [file Table_1.DOCX]

| Supplementary table 1. | | | | |
| --- | --- | --- | --- | --- |
| Univariate and multivariate COX regression analysis of progression-free survival in training group. | | | | |
| Characteristics | Univariate | | Multivariate | |
|  | Hazard Ratio（95%CI） | p Value | Hazard Ratio（95%CI） | p Value |
| Histology (LUSC vs. LUAD) | 0.951(0.769-1.175) | 0.641 | - | - |
| Gender (female vs. male) | 0.897(0.709-1.135) | 0.366 | - | - |
| Age (<63 vs. ≥63) | 0.742(0.601-0.918) | 0.006 | 0.685(0.547-0.857) | 0.001 |
| Primary site |  |  |  |  |
| Upper lobe | 1 | 0.003 | 1 | 0.056 |
| Middle lobe | 0.891(0.534-1.489) | 0.660 | 1.239(0.732-2.098) | 0.424 |
| Lower lobe | 1.176(0.935-1.478) | 0.165 | 1.174(0.93-1.483) | 0.176 |
| Hilus of the lung | 1.87(1.198-2.917) | 0.006 | 1.3(0.794-2.129) | 0.297 |
| Overlapping lesion of lung | 0.457(0.233-0.893) | 0.022 | 0.448(0.219-0.917) | 0.028 |
| Laterality (right vs. left) | 1.013(0.82-1.251) | 0.907 | - | - |
| Grade |  |  |  |  |
| Well differentiated | 1 | <0.001 | 1 | 0.098 |
| Moderately differentiated | 2.832(1.68-4.775) | <0.001 | 1.744(1.01-3.012) | 0.046 |
| Poorly and undifferentiated | 2.718(1.575-4.691) | <0.001 | 1.536(0.865-2.729) | 0.143 |
| TNM stage |  |  |  |  |
| I | 1 | <0.001 | 1 | <0.001 |
| II | 2.175(1.602-2.954) | <0.001 | 1.802(1.299-2.499) | <0.001 |
| III+IV | 3.754(2.835-4.972) | <0.001 | 3.048(2.226-4.173) | <0.001 |
| Scope of surgery |  |  |  |  |
| Sublobectomy | 1 | 0.053 | 1 | 0.775 |
| Lobectomy | 1.177(0.824-1.681) | 0.371 | 0.948(0.656-1.369) | 0.775 |
| Extended lobectomy | 1.313(0.841-2.052) | 0.231 | 1.011(0.627-1.63) | 0.965 |
| Pneumonectomy | 1.757(1.127-2.739) | 0.013 | 1.154(0.7-1.902) | 0.575 |
| Smoking index |  |  |  |  |
| 0 | 1 | 0.070 | 1 | 0.436 |
| <387.5 | 0.986(0.644-1.51) | 0.948 | 0.748(0.481-1.163) | 0.198 |
| >387.5 | 1.285(1.021-1.616) | 0.032 | 0.939(0.732-1.205) | 0.623 |
| Adjuvant therapy (yes vs. none) | 1.578(1.273-1.957) | <0.001 | 1.205(0.963-1.509) | 0.103 |
| Pyrexia before surgery (yes vs. no) | 1.081(0.805-1.452) | 0.605 | - | - |
| Pyrexia after surgery (yes vs. no) | 0.731(0.591-0.904) | 0.004 | 0.839(0.673-1.046) | 0.118 |
| Hyperpyrexia before surgery (yes vs. no) | 1.095(0.653-1.837) | 0.732 | - | - |
| Hyperpyrexia after surgery (yes vs. no) | 1.016(0.791-1.304) | 0.903 | - | - |
| VATS (yes vs. no) | 0.413(0.292-0.584) | <0.001 | 0.74(0.506-1.082) | 0.121 |
| ANRI (<4.91 vs. ≥4.91) | 1.412(1.143-1.745) | 0.001 | - | - |
| NLR (<2.745 vs. ≥2.745) | 0.762(0.614-0.946) | 0.014 | - | - |
| AGR (<1.40 vs. ≥1.40) | 1.306(1.055-1.617) | 0.014 | 1.132(0.906-1.414) | 0.276 |
| SII (<572.21 vs. ≥572.21) | 0.751(0.608-0.928) | 0.008 | - | - |
| SIRI (<1.155 vs. ≥1.155) | 0.772(0.625-0.954) | 0.017 | - | - |
| NPS |  |  |  |  |
| 0 | 1 | 0.732 | - | - |
| 1 | 0.901(0.612-1.326) | 0.596 | - | - |
| 2 | 1.331(0.496-3.569) | 0.570 | - | - |
| F-NLR |  |  |  |  |
| 0 | 1 | 0.001 | - | - |
| 1 | 1.304(1.012-1.679) | 0.040 | - | - |
| 2 | 1.617(1.25-2.092) | <0.001 | - | - |
| SIS |  |  |  |  |
| 0 | 1 | 0.253 | - | - |
| 1 | 1.204(0.936-1.549) | 0.149 | - | - |
| 2 | 1.245(0.93-1.667) | 0.142 | - | - |
| AFR (<10.625 vs. ≥10.625) | 1.429(1.151-1.775) | 0.001 | - | - |
| PNI (<50.925 vs. ≥50.925) | 1.354(1.094-1.677) | 0.005 | - | - |
| ALRI (<14.75 vs. ≥14.75) | 1.127(0.892-1.423) | 0.317 | - | - |
| MLR (<0.345 vs. ≥0.345) | 0.78(0.622-0.978) | 0.031 | - | - |
| BLR (<0.00675 vs. ≥0.00675) | 0.637(0.502-0.807) | <0.001 | 0.694(0.546-0.883) | 0.003 |
| PLR (<168.745 vs. ≥168.745) | 0.777(0.616-0.981) | 0.034 | - | - |
| GLR (<38.365 vs. ≥38.365) | 1.256(0.781-2.018) | 0.347 | - | - |
| FIB-4 score (<1.285 vs. ≥1.285) | 0.948(0.768-1.171) | 0.621 | - | - |
| GAPI (<0.123 vs. ≥0.123) | 1.214(0.964-1.528) | 0.099 | - | - |
| APRI (<0.072 vs. ≥0.072) | 1.337(1.074-1.665) | 0.009 | 1.071(0.854-1.345) | 0.552 |
| Abbreviation: LUAD, lung adenocarcinoma, LUSC, lung squamous cell carcinoma; ANRI, aspartate transaminase-to-neutrophil ratio index; NLR, neutrophil-to-lymphocyte ratio; AGR, albumin-to-globulin ratio; SII, systemic immune inflammation index; SIRI, systemic inflammation response index; NPS, neutrophil-platelet score; F-NLR, fibrinogen-NLR score; SIS, systemic inflammation score; AFR, albumin-to-fibrinogen ratio; PNI, prognostic nutrition index; ALRI, aspartate transferase (AST)-to-lymphocyte ratio; MLR, monocyte-to-lymphocyte ratio; BLR, basophil-to-lymphocyte ratio; PLR, platelet-to-lymphocyte ratio; GLR, glutamyl transpeptidase (GGT)-to-lymphocyte ratio; FIB-4, fibrosis index based on four factors; GAPI, glutamyl transpeptidase (GGT)-to-platelet ratio; APRI, aspartate aminotransferase-to-platelet ratio index. | | | | |
