# Supplementary material for: The Prognostic Value of Multiple Systemic Inflammatory Biomarkers in Preoperative Patients With Non-small Cell Lung Cancer
Source: Front Surg. 2022 Apr 4;9:830642. doi: 10.3389/fsurg.2022.830642 (PMC9013845; doi:10.3389/fsurg.2022.830642)
Supplement: Supplementary Table 2 — Univariate and multivariate COX regression analysis of overall survival in training group. [file Table_2.DOCX]

| Supplementary table 2. | | | | |
| --- | --- | --- | --- | --- |
| Univariate and multivariate COX regression analysis of overall survival in training group. | | | | |
| Characteristics | Univariate | | Multivariate | |
|  | Hazard Ratio（95%CI） | p Value | Hazard Ratio（95%CI） | p Value |
| Age (<63 vs. ≥63) | 0.635(0.515-0.782) | <0.001 | 0.572(0.464-0.705) | <0.001 |
| Grade |  |  |  |  |
| Well differentiated | 1 | <0.001 | 1 | 0.010 |
| Moderately differentiated | 3.073(1.757-5.373) | <0.001 | 2.141(1.214-3.776) | 0.009 |
| Poorly and undifferentiated | 3.707(2.087-6.585) | <0.001 | 2.215(1.233-3.981) | 0.008 |
| TNM stage |  |  |  |  |
| I | 1 | <0.001 | 1 | <0.001 |
| II | 2.125(1.57-2.876) | <0.001 | 2.064(1.517-2.808) | <0.001 |
| III+IV | 3.762(2.85-4.965) | <0.001 | 3.647(2.745-4.845) | <0.001 |
| Albumin (g/L) (<42.65 vs. ≥42.65) | 1.334(1.07-1.662) | 0.010 | - | 0.245 |
| Globulin (g/L) (<28.25 vs. ≥28.25) | 0.761(0.616-0.94) | 0.011 | - | 0.051 |
| Lymphocyte (10^9^/L ) (<1.485 vs. ≥1.485) | 1.249(0.998-1.562) | 0.052 | - | 0.157 |
| Basophil (10^9^/L) (<0.015 vs. ≥0.015) | 0.811(0.657-1) | 0.050 | - | 0.173 |
|  | | | | |
